# Features of the ancestral bilaterian inferred from *Platynereis dumerilii *ParaHox genes

**DOI:** 10.1186/1741-7007-7-43

**Published:** 2009-07-23

**Authors:** Jerome HL Hui, Florian Raible, Natalia Korchagina, Nicolas Dray, Sylvie Samain, Ghislaine Magdelenat, Claire Jubin, Béatrice Segurens, Guillaume Balavoine, Detlev Arendt, David EK Ferrier

**Affiliations:** 1 Department of Zoology, University of Oxford, Oxford, UK; 2 EMBL, 69117 Heidelberg, Germany; 3 MFPL, Vienna, Austria; 4 CNRS-CGM, Gif-sur-Yvette, France; 5 Génoscope, Centre National de Séquençage, Evry, France; 6 The Scottish Oceans Institute, University of St Andrews, St Andrews, UK; 7 Faculty of Life Sciences, Michael Smith Building, University of Manchester, Manchester, UK; 8 Department of Molecular, Cellular and Developmental Biology, Yale University, New Haven, USA; 9 Institut Jacques Monod, UMR 7592 CNRS/Université Denis Diderot – Paris VII, Paris, France

## Abstract

**Background:**

The ParaHox gene cluster is the evolutionary sister to the Hox cluster. Whilst the role of the Hox cluster in patterning the anterior-posterior axis of bilaterian animals is well established, and the organisation of vertebrate Hox clusters is intimately linked to gene regulation, much less is known about the more recently discovered ParaHox cluster. ParaHox gene clustering, and its relationship to expression, has only been described in deuterostomes. Conventional protostome models (*Drosophila melanogaster *and *Caenorhabditis elegans*) are secondarily derived with respect to ParaHox genes, suffering gene loss and cluster break-up.

**Results:**

We provide the first evidence for ParaHox gene clustering from a less-derived protostome animal, the annelid *Platynereis dumerilii*. Clustering of these genes is thus not a sole preserve of the deuterostome lineage within Bilateria. This protostome ParaHox cluster is not entirely intact however, with *Pdu-Cdx *being on the opposite end of the same chromosome arm from *Pdu-Gsx *and *Pdu-Xlox*. From the genomic sequence around the *P. dumerilii *ParaHox genes the neighbouring genes are identified, compared with other taxa, and the ancestral arrangement deduced.

**Conclusion:**

We relate the organisation of the ParaHox genes to their expression, and from comparisons with other taxa hypothesise that a relatively complex pattern of ParaHox gene expression existed in the protostome-deuterostome ancestor, which was secondarily simplified along several invertebrate lineages. Detailed comparisons of the gene content around the ParaHox genes enables the reconstruction of the genome surrounding the ParaHox cluster of the protostome-deuterostome ancestor, which existed over 550 million years ago.

## Background

The ParaHox gene cluster was first discovered in the invertebrate chordate amphioxus (*Branchiostoma floridae*) [1]. ParaHox gene clustering is conserved in humans and other tetrapods, but disrupted in several other chordates [2-4]. The ancestral condition for the chordates is, however, clearly one of possession of a ParaHox cluster, which has been conserved since the Cambrian along both the cephalochordate and tetrapod lineages.

The organisation of the cluster and the phylogenetic relationships of its component genes (*Gsx*, *Xlox *and *Cdx*) relative to the Hox cluster genes are consistent with a paralogous relationship between the ParaHox and Hox clusters, that is, they are evolutionary sisters. Whilst Hox gene clustering is widespread across the bilaterians, the ParaHox cluster has only been found in chordates thus far (as well as one example from the probable sister group to the bilaterians, the cnidarians[5]). In protostomes that have been examined to date (insects and nematodes) the ParaHox cluster does not exist, and one or more ParaHox genes have been lost. This ParaHox gene loss is clearly a secondarily derived condition for protostomes, since all three ParaHox genes are present in a variety of lophotrochozoan protostomes, including annelids and molluscs [6-9]. Whilst the ancestral presence of all three ParaHox genes is now well established for protostomes, the genomic organisation of the genes in an animal that is not as derived as insects and nematodes, and which still retains all three genes, has not been determined.

The ordered clustering of the Hox genes is related to their expression and function, at least in the vertebrates. The order of the genes along the chromosome corresponds to the order of the gene expression domains along the embryonic anterior-posterior axis: the phenomenon of colinearity. Due to the paralogous relationship and retention of clustering between the ParaHox and Hox cluster, there is a distinct possibility that the organisation of the ParaHox cluster also relates to the expression and function of the component genes in a similar fashion to the Hox cluster situation.

ParaHox gene organisation and expression has been widely examined within deuterostomes. The prototypical ParaHox cluster of amphioxus exhibits spatial colinearity, with *AmphiGsx *expressed in the anterior central nervous system (CNS), *AmphiXlox *in a more central region of the CNS and the developing gut, and *AmphiCdx *at the posterior end of the larva in both the CNS and gut [1,10]. This has distinct similarities to vertebrate ParaHox gene expression. Vertebrate Gsx genes (usually called *Gsh1 *and *Gsh2*) have anterior boundaries of expression in the brain with extensive expression posteriorly into the neural tube, in a dorso-ventrally restricted fashion that may be comparable to *Drosophila *[11-17]. Vertebrate Xlox genes (with synonyms of *PDX1*, *IPF1*, *IDX1*, *XlHbox8*, *STF1 *or *MODY4*) are expressed in the gut during pancreas development [18-22] and in the CNS [23-25]. Vertebrate Cdx genes are predominantly posterior patterning genes, expressed in the CNS, mesoderm and gut [25-28]. In invertebrate deuterostomes, apart from amphioxus, the ParaHox cluster has broken apart [2,29] but there are still elements of the spatial restriction and tissue specificity of ParaHox expression. In urochordates Gsx is expressed in a small domain in the anterior CNS [30], Xlox (called *Ci-IPF1*) is in mesenchymal cells and some cells of the CNS [31], and Cdx patterns the posterior tadpole tail and is expressed in the hindgut of post-metamorphic animals [32,33]. In the echinoderm, *Strongylocentrotus purpuratus*, Gsx is expressed in a small patch of putative nerve cells, whilst Xlox and Cdx have staggered expression domains in the posterior gut tube [29], and in a starfish, *Archaster typicus*, Xlox is expressed throughout the early archenteron and a few vegetal ectodermal cells [34]. In summary, deuterostome Gsx tends to be expressed solely in the CNS with a rostral anterior limit, Xlox is expressed both in the CNS and the developing gut, in central regions such as the pancreas of vertebrates, and Cdx is expressed in more posterior regions of the CNS and gut. Whether the deuterostome ParaHox genes exist in an intact or broken cluster may depend on the regulatory mechanism(s) controlling the temporal activation of the genes [2,35].

ParaHox gene expression has been more sparsely sampled in protostomes, apart from Cdx (or caudal). Gsx expression has been documented in the insects *Drosophila *and *Tribolium*, and the polychaetes *Capitella *and *Nereis virens*. Insect Gsx is expressed along a pair of medio-laterally restricted neural columns, and has a role in neuronal patterning [11,36]. There are also domains of expression in the head region of these insects that have yet to be fully characterised, but do include expression in neural cells [11,36,37]. In contrast, expression of Gsx in the polychaete *Capitella *is restricted to a small domain close to the anterior end of the CNS [7]. This is very different to the spatially and temporally dynamic expression of Gsx in the nereid polychaetes, *Nereis virens *[38] and *Platynereis dumerilii *(described below). The central ParaHox gene, Xlox, is missing from all ecdysozoan genomes sequenced to date, but is present in lophotrochozoans. In the leeches *Helobdella triserialis *and *Hirudo medicinalis *Xlox (named *HtrA2 *or *Lox3*) is expressed throughout the midgut, as is also the case for the polychaete *Capitella *[7,39,40]. No neural Xlox expression has been described in these annelids. Nereid Xlox expression also has a midgut component, but in contrast to these other annelids is also expressed in the CNS [38] (and see below). In contrast to the sparse data on protostome Gsx and Xlox expression, Cdx has been examined in a large variety of taxa. First characterised as a posterior patterning gene acting early in the segmentation gene cascade in *Drosophila *(in which the gene is called *caudal*) [41], Cdx has subsequently been studied in many other arthropods [42-51]. Broadly, Cdx is a posterior patterning gene in all of these animals, as it also is in the nematode *Caenorhabditis elegans *(where the gene is called *pal-1*) [52] and the mollusc, *Patella *[53]. In the annelids *Platynereis *([54] and herein), *Nereis *[38], *Tubifex *[55] and *Capitella *[7] there are both anterior and posterior expression domains of Cdx (see Discussion). There is little data on the genomic organisation of protostome ParaHox genes and how it may relate to their expression.

Here we provide the first description of the expression patterns for all three ParaHox genes for a protostome animal in relation to their genomic organisation, in the polychaete *P. dumerilii*. Clustering of protostome ParaHox genes is shown for the first time, which reveals that some clustering of ParaHox genes has been conserved on both the protostome and deuterostome lineages. The *P. dumerilii *ParaHox cluster is not, however, entirely intact. The posterior member, *Pdu-Cdx*, has been separated from the other two genes, *Pdu-Gsx *and *Pdu-Xlox*, and the two parts of the *Platynereis *ParaHox cluster now reside on opposite ends of the same chromosome arm. Comparison of the genes neighbouring the *Platynereis *ParaHox genes with the map positions of the mammalian orthologues allows the reconstruction of the genomic region surrounding the ParaHox cluster of the protostome-deuterostome ancestor (PDA), an extinct animal that lived over 550 million years ago. The details of the *Platynereis *ParaHox gene expression patterns, by comparison with those of other animals, imply a complex role for Gsx in the PDA's CNS and a possible function in protostome mouth development, a role for Xlox in CNS patterning as well as gut development, and a complex and dynamic pattern of Cdx expression in polychaetes that correlates with the relocation of the gene out of the cluster.

## Results

### ParaHox gene sequences

**Figure 1:**
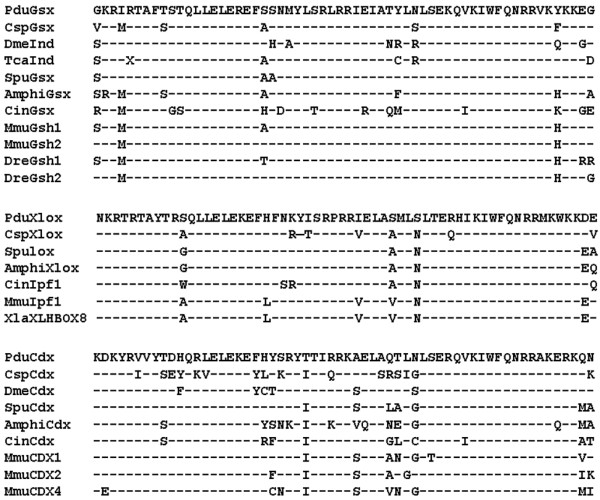
**Alignments of *P. dumerilii *ParaHox homeodomains to bilaterian orthologs**. *Platynereis *sequences are at the top of the alignments. Dashes denote sequence identities. Abbreviations: Amphi, cephalochordate *Branchiostoma floridae*; Cin, urochordate *Ciona intestinalis*; Csp, polychaete *Capitella sp.I*; Dme, fruit fly *Drosophila melanogaster*; Dre, zebrafish *Danio rerio*; Mmu, mouse *Mus musculus*; Spu, echinoderm *Strongylocentrotus purpuratus*; Tca, beetle *Tribolium castaneum*; Xla, amphibian *Xenopus laevis*.

The entire coding sequences for all three *P. dumerilii *ParaHox genes was isolated by a combination of degenerate primer PCR, RACE PCR and sequencing of bacterial artificial chromosome (BAC) genomic clones (see Methods). The main region of conservation between ParaHox genes is in the homeobox. Alignments of each *P. dumerilii *ParaHox homeodomain to representatives from other animals are shown in Figure 1. The clear classification of the *P. dumerilii *ParaHox genes into their orthology groups is apparent from phylogenetic analyses (Figure S1 in Additional file 1). Other conserved domains besides the homeodomains are the N-terminal SNAG transcriptional repressor domain in Gsx, and the hexapeptide motifs just upstream of the homeodomains in both Xlox and Cdx (Additional file 1, Figure S2 to S4).

### ParaHox gene organisation

**Figure 2:**
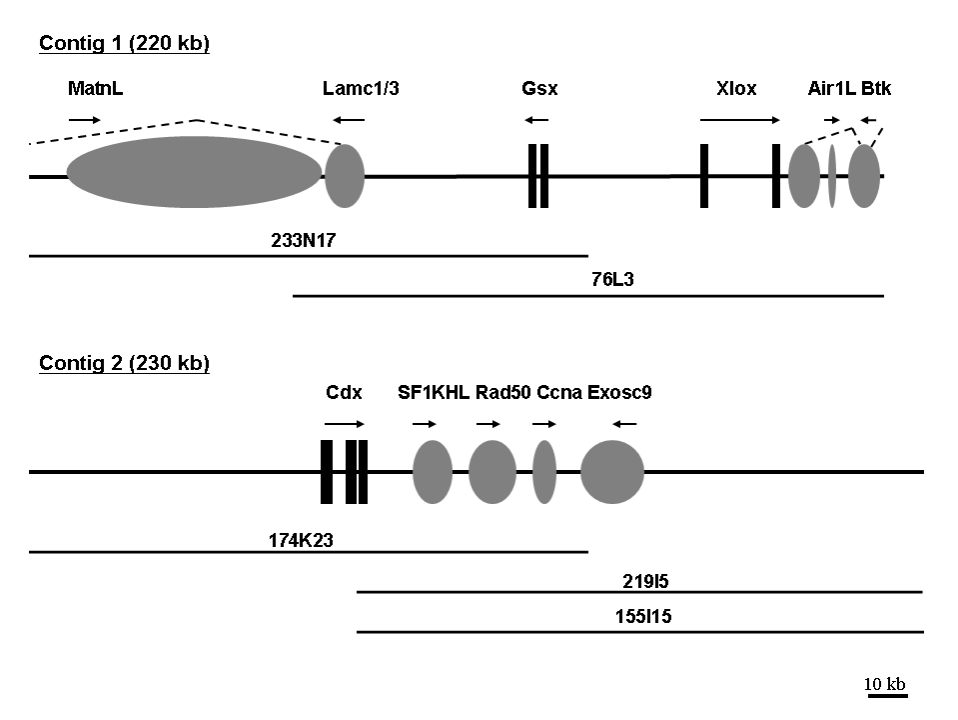
***P. dumerilii *ParaHox gene genomic contigs**. Fully sequenced BAC clones are shown with clone identity numbers. Black boxes are homeobox gene exons, and grey ovals are non-homeobox genes. Arrows denote transcriptional orientation.

Both *Pdu-Gsx *and *Pdu-Xlox *are located on a single BAC clone, 42 kb apart, and are organised in a head-to-head orientation (Figure 2). This is in contrast to the tandem orientation of Gsx and Xlox in chordates [56]. *Pdu-Cdx *is not on this *Pdu-Gsx/Pdu-Xlox *BAC clone. Several independent BAC clones containing *Pdu-Cdx *were isolated, and sequencing of these clones revealed no overlap of the *Pdu-Cdx *BACs with the *Pdu-Gsx/Pdu-Xlox *BAC. Complete sequencing of the ParaHox contigs identified no other homeobox-containing genes besides *Pdu-Gsx*, *Pdu-Xlox *and *Pdu-Cdx *(Figure 2). Although there is a putative partial reverse-transcriptase-like gene found between *Pdu-Gsx *and *Pdu-Xlox*, it is likely to be the remains of a transposable element. We conclude that there are no internal non-homeobox genes between *Pdu-Gsx *and *Pdu-Xlox*. Such a situation is analogous to that reported for intact chordate ParaHox clusters [56].

**Figure 3:**
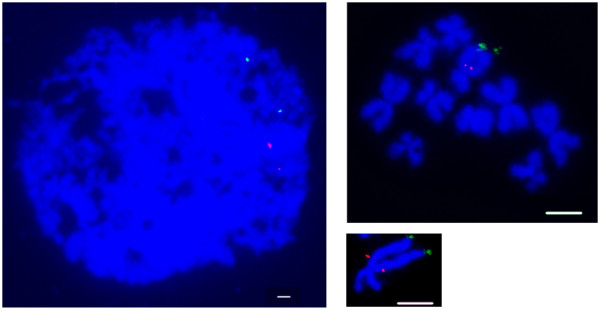
**Fluorescent *in situ *hybridisation to *P. dumerilii *chromosomes**. The left-hand panel shows signals of *Pdu-Gsx *and *Pdu-Xlox *(CHORI305_76L3) (red) and *Pdu-Cdx *(CHORI305_108J4) (green) on a typical interphase nucleus, showing that the red and green signals are consistently in similar regions of the nucleus, but with a relatively large distance between them. This is consistent with results on metaphase chromosomes (right-hand panels), in which signals of *Pdu-Gsx *(CHORI305_233N17) (red) are located near to the centromere of a q arm, and *Pdu-Cdx *(CHORI305_108J4) (green) on the telomeric end on the same chromosome. (Bars = 20 μm).

Two-colour chromosomal fluorescent *in situ *hybridisation (FISH) with *Pdu-Gsx*, *Pdu-Gsx/Pdu-Xlox *and *Pdu-Cdx *BAC clones on *Platynereis *metaphase chromosome spreads revealed that *Pdu-Gsx *and *Pdu-Xlox *are located at the centromeric end of one of the metacentric chromosomes of *Platynereis*, whilst *Pdu-Cdx *is located close to the telomere on the same chromosome arm (Figure 3).

### ParaHox gene expression

Only a partial account of *Platynereis *ParaHox gene expression has been available until now, including the expression of *Pdu-Cdx *during development and regeneration and of *Pdu-Gsx *at 48 hours post fertilisation (hpf) [54,57]. In order to compare the expression of all three ParaHox genes in *Platynereis*, we performed a series of whole-mount *in situ *hybridisations (WMISH) during larval development.

**Figure 4:**
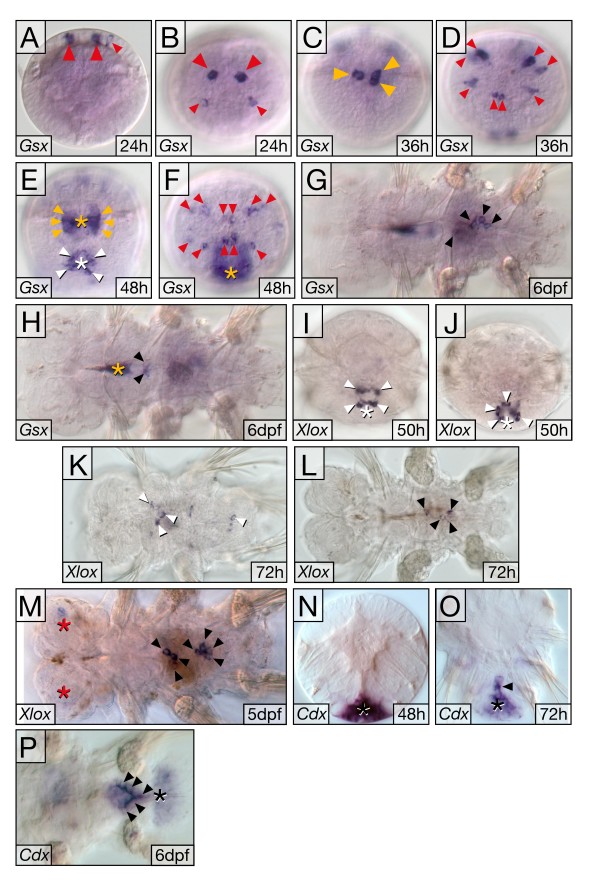
**Expression of *Pdu-Gsx, Pdu-Xlox *and *Pdu-Cdx *during larval development**. **(A-F) ***Pdu-Gsx *is expressed in distinct cell clusters in the apical hemisphere (red arrowheads), and stomodeum (yellow arrowheads) that marks the anterior end of the future gut. Expression is also found in ventral neuroectoderm (E, white arrowheads). **(G, H) **Later, *Pdu-Gsx *marks specific cells (black arrowheads) in midgut (G) and posterior foregut (H). **(I-K) **Early *Pdu-Xlox *expression is confined to cells in the medial ventral plate (I, J; white arrowheads), where weak expression also persists to later stages (K). **(L, M) **In parallel, *Pdu-Xlox *transcripts appear inside the larvae (black arrowheads), marking distinct cell clusters in prospective midgut. Weak expression is also observed in bilateral lobes of the larval brain (red asterisks). **(N-P) **In trochophore larva (N), *Pdu-Cdx *is prominent in the proctodeal area (black asterisk); which subsequently (O, P), resolves into cells of the posterior midgut and hindgut (black arrowheads), and pygidial epidermis (black asterisks). For *Pdu-Gsx*, we also observe signal in the anterior foregut region (yellow asterisk in H), but we presently cannot rule out that this anterior signal results from probe trapping in the foregut cavity rather than being a further *Pdu-Gsx *expression domain. Orientation of panels: (A, C, E, I, N, O) ventral views, apical/anterior to the top; (B, D, F, J) apical views, ventral to the bottom; (G, H, K-M, P) ventral views, anterior to the left. Asterisks mark stomodeum/foregut cavity (yellow), ventral plate (white), head lobes (red) and proctodeum/pygidium (black), respectively.

*Pdu-Gsx *expression is very dynamic, and transcripts are already detected by 24 hpf by WMISH. Expression is seen in a few cells in the apical hemisphere (Figure 4A and 4B), where *Pdu-Gsx*-positive cells continue to be observed throughout trochophore stages, both in the developing apical organ and cerebral ganglia (Figure 4D and 4F). The flask-shaped appearance of *Pdu-Gsx*-positive cells, with long sensory dendrites (data not shown), and their position in the medial forebrain anlage is consistent with them being sensory-neurosecretory cells [58]. As reported previously, *Pdu-Gsx *expression is also seen in the ventral plate of 48 hpf larvae during differentiation of the trunk CNS [57]. Besides the expression domains in prospective neural tissue, *Pdu-Gsx *transcripts are also detected in the stomodeum, where they appear prior to 36 hpf in two bilateral cell clusters (Figure 4C). Subsequently stomodeal expression becomes more prominent, and forms two bilateral stripes at 48 hpf (Figure 4E). At 6 days of larval development *Pdu-Gsx *expression is most prominent in small cell clusters of the cellularised gut, both in the region of the midgut and the posterior foregut (Figure 4G and 4H).

*Pdu-Xlox *transcription appears to be initiated considerably later than *Pdu-Gsx *expression. Whilst no expression is detectable at 24 or 36 hpf by WMISH, *Pdu-Xlox *transcripts appear in a group of cells in the medial ventral plate of the metatrochophore larvae by 50 hpf (Figure 4I and 4J). Intriguingly, this expression is very similar in appearance to the medial ventral plate expression of *Pdu-Gsx *at a similar stage of development (Figure 4E). Expression in the medial ventral plate is also observed at 72 hpf (Figure 4K). At this stage *Pdu-Xlox *expression is also initiated in the midgut rudiment (Figure 4L). By 5 days of development *Pdu-Xlox *transcripts are confined to two separate clusters of cells in the anterior and posterior midgut (Figure 4M). At this stage neural expression is still clearly visible in a bilateral pair of spots in the cerebral ganglia (Figure 4M).

Clear *Pdu-Cdx *expression is evident by 15 hpf [54]. The ectodermal expression encircles the entire proctodeal portion of the blastopore and extends anteriorly along the edges of the blastopore up to the posterior side of the stomodeum. There are two horns of *Pdu-Cdx *expression deeper inside the larvae at 15 to 20 hpf, which are possibly mesodermal or endodermal precursors. By 36 hpf the blastopore has completely separated into stomodeum and proctodeum, and *Pdu-Cdx *expression is confined to the proctodeum and a small cap of posterior ectoderm ([54]; Figure 4N). As development proceeds through to the 6-day-old three-segment larvae the proctodeal expression resolves into midgut and hindgut expression and the posterior ectodermal expressing cells become pygidium epidermis (Figure 4P).

### Neighbouring genes

A contig of 220 kb was sequenced around *Pdu-Gsx *and *Pdu-Xlox*, and a separate contig of 230 kb was sequenced around *Pdu-Cdx*. There are several flanking genes outside the ParaHox genes in the two contigs (Figure 2). Two genes are found next to *Pdu-Gsx*, and two genes on the other side next to *Pdu-Xlox*, and four genes flanking one side of *Pdu-Cdx*. The other side flanking *Pdu-Cdx *contains a region of repetitive sequence with putative transposable elements and repeats. The characteristics of these neighbouring genes, and the rationale for their naming, is given in Additional file 1.

The two putative genes outside the annelid ParaHox cluster at the *Pdu-Gsx *end are Matrilin-like (*Pdu-MatnL*) and Laminin Gamma (*Pdu-Lamc1/3*) (Figure 2 and Additional file 1, Figures S5 and S6). These two genes lie around 92 kb and 44 kb away from *Pdu-Gsx*, respectively (calculating from the start methionine codon of each gene). It seems that *Pdu-MatnL *lies within a large intron of *Pdu-Lamc1/3*, with the 3' exon(s) of *Pdu-Lamc1/3 *lying beyond the reach of the present contig. On the other side of the *Pdu-Gsx*/*Pdu-Xlox *contig, adjacent to *Pdu-Xlox*, lie *Pdu-AIR1-Like (Pdu-AIR1L) *and *Pdu-Btk *(Figure 2 and Additional file 1, Figures S7 and S8). *Pdu-AIR1L *is around 32 kb away from the start methionine codon of *Pdu-Xlox*. Two regions of sequence similarity to tyrosine kinases are found at around 24 kb and 36 kb away from the start methionine codon of *Pdu-Xlox*, separated by *Pdu-AIR1L*, which is transcribed in the opposite orientation (Figure 2), and together these tyrosine kinase regions constitute a section of the *Pdu-Btk *gene. From the sequence alignments we suppose that the N-terminal end of *Pdu-Btk *is encoded by exons that lie beyond the present contig (Figure 2 and Additional file 1, Figure S8). In the *Pdu-Cdx *contig, there are four putative genes on the 3' side of *Pdu-Cdx*: *Pdu-SF1KH-Like (Pdu-SF1KHL)*, *Pdu-Rad50*, *Pdu-Ccna*, and *Pdu-Exosc9 *(Additional file 1, Figures S9 to S12), with putative start methionine codons located around 30 kb, 46 kb, 64 kb and 86 kb away from the start methionine codon of *Pdu-Cdx*, respectively.

### Synteny

**Figure 5:**
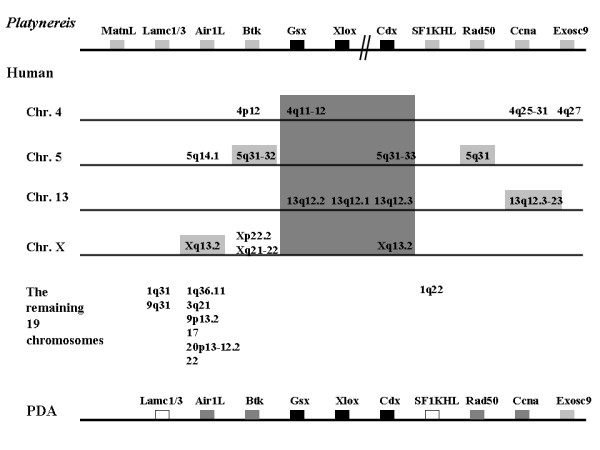
**Ortholog locations of the *P. dumerilii *ParaHox neighbouring genes in human**. The top line shows a merge of the *Platynereis *contigs, black boxes are the ParaHox genes, and other boxes are neighbouring genes. Gene order is not biologically significant and the cluster break is denoted by double-parallel lines. The middle panel shows the locations of the human orthologs. The central large grey shaded area is the location of the ParaHox genes, and other grey shaded boxes are orthologs that are tightly linked to the human ParaHox genes. The bottom line shows probable genomic organisation around the ParaHox genes in the protostome-deuterostome ancestor, but the ordering of the genes around the ParaHox cluster is not significant in this figure, and cannot be deduced from this data. Black boxes represent homeobox genes, and ParaHox neighbours are ranked according to support with dark grey boxes being the most strongly supported, followed by light grey box and white boxes (see text for details).

Some of the genes neighbouring the ParaHox clusters of chordates were clearly flanking the cluster in the chordate ancestor [4,56,59]. Given the possibility of detecting such ancient synteny, the genomic locations of human orthologues to the *P*.*dumerilii *flanking genes were compared, and are shown in Figure 5. Interestingly, neighbours of the ParaHox genes in the protostome *P*.*dumerilii *can also be found close to the ParaHox genes in human.

Figure 5 shows the reconstruction of the ParaHox synteny region of the PDA. Humans have four ParaHox regions for consideration, one of which contains the complete ParaHox cluster (on chromosome 13) and three that contain the remains of the degenerate clusters that have undergone ParaHox gene loss (chromosomes 4, 5 and X) [56]. Figure 5 shows the genomic locations of the human orthologues of the *Platynereis *ParaHox flanking genes (considering both *Platynereis *ParaHox contigs together). It is clear that one or more of the paralogues of each gene under consideration is located on the same chromosome as a ParaHox cluster for several of these genes (including both the complete and degenerate human clusters), and in some cases is adjacent to a ParaHox locus. A human Btk gene (*ITK*) has a tight linkage to the ParaHox cluster at 5q31, and two further pairs of paralogues (*TEC*, *TXK*, and *BMX*, *BTK*) show looser linkage to the cluster on chromosome 4 and X (Figure 5). One of the human AIR-like genes (*ZCCHC13*) is close to the ParaHox region on the X chromosome at Xq13.2, and a second Air1L gene (*ZCCHC9*) is more loosely linked to the ParaHox locus on human chromosome 5 (Figure 5). Human *Rad50 *is adjacent to the ParaHox region on human chromosome 5, at 5q31, and the human Ccna gene (*CCNA1*) sits next to the ParaHox cluster at 13q12.3-23, with a paralogue (*CCNA2*) also loosely linked to the ParaHox region on chromosome 4. These gene families then (Btk, AIR1-like, Ccna and Rad50) have a close genomic association with the ParaHox genes in both *Platynereis *and humans, and so we hypothesise that they were close neighbours in the bilaterian ancestor.

The remaining genes may well have also been within this ancient ParaHox synteny region, but the data in support of this conclusion is not as strong as that for Btk, AIR1-like, Ccna and Rad50. An EXOSC9 gene is loosely linked to the human ParaHox region on chromosome 4. This is close to a paralogue of CCNA (*CCNA2 *at 4q25-31) whose evolutionary sister (*CCNA1*) is tightly linked to the 13q12.1-3 ParaHox locus. Laminin Gamma and SF1KHL genes are not linked to ParaHox genes in humans; however, they are linked to each other on human chromosome 1 (Figure 5). The large size of the Matrilin-like family of genes in humans means that it is difficult to draw meaningful conclusions about orthology relationships between the *Platynereis *gene described here (*Pdu-MatnL*) and particular human Matrilin-like genes and their genomic locations. Whilst the Matrilin-like genes cannot thus be included in the ParaHox syntenic region reconstruction, the Exosc9, Laminin Gamma and SF1KHL genes would only require single translocation events along the human lineage to be accommodated, to allow them to be included as potential ParaHox neighbours (Figure 5).

## Discussion

### ParaHox gene clustering

The existence of colinearity in the ParaHox cluster of chordates implies that the organisation of the ParaHox genes could well be linked to their control and operation. If there is some form of long-range regulatory mechanism operating across the ParaHox genes, as clearly happens with the vertebrate Hox genes [60], then the organisation of the genome around the ParaHox genes would be expected to be refractory or intolerant to extensive rearrangements during evolution, as has been observed for genomic regulatory blocks (GRBs) in vertebrate genomes [61]. Detection of common gene neighbours in distinct animal lineages also implies conserved synteny inherited from the last common ancestor. Regions of these ancestral genomes can thus be reconstructed even though the organisms in which they existed have long since gone extinct. Such genome reconstructions have the potential to help us understand the ancestral regulatory mechanisms operating on key developmental control genes, such as clusters of homeobox genes.

**Figure 6:**
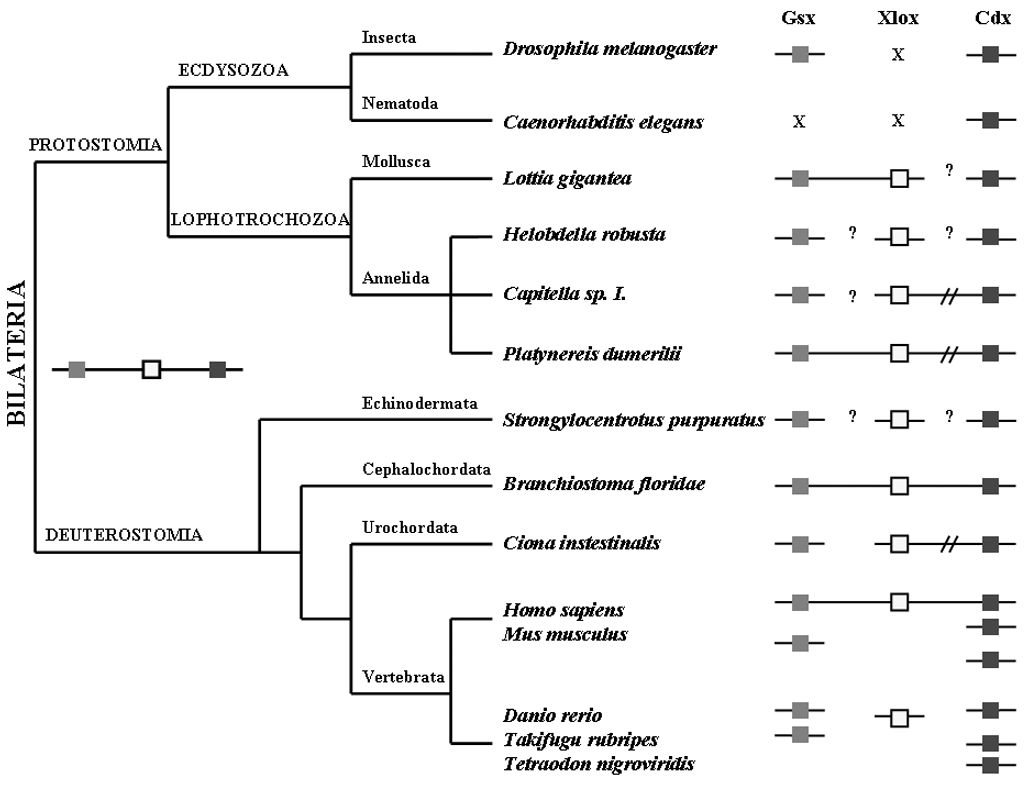
**Summary of bilaterian ParaHox gene organisation**. Boxes indicate ParaHox genes. Boxes on the same line represent genes linked on the same chromosome. Cluster breaks with intervening non-homeobox genes are denoted by double-parallel lines. Gene losses are denoted by crosses. Question marks indicate no information available for the chromosomal location. However, it should be noted that there are flanking non-homeobox genes on both sides of all the ParaHox orthologues with question marks, except for one side of both Gsx and Xlox in sea urchin *Strongylocentrotus purpuratus*, in which these two ParaHox genes are located at the ends of their respective scaffolds.

Until now the ParaHox cluster has only been found in chordates within the bilaterians. The close linkage of *Pdu-Gsx *and *Pdu-Xlox*, with no intervening genes, is the first example of ParaHox gene clustering to be found in a protostome (Figure 6). This is perhaps surprising given the supposed paralogous nature of the ParaHox cluster with the Hox cluster, which is usually assumed to be clustered quite widely across the animal kingdom and exhibit a link between the organisation of the genes in the Hox cluster and their regulation (colinearity). There are, however, an increasing number of examples of Hox clusters that have broken apart, and the underlying mechanistic basis for Hox cluster maintenance or disintegration is unclear (reviewed in [35,60,62]). As further Hox and ParaHox clusters are characterised from a greater diversity of taxa, and the regulatory mechanisms operating within the clusters are elucidated, we will gain a much deeper understanding of the role of these key developmental control genes in animal development and evolution, and in particular whether clustering of these genes reflects mechanistic constraints or simply evolutionary inertia.

### ParaHox expression in the protostome-deuterostome ancestor

Comparisons between *Platynereis *gene expression and the orthologues of deuterostomes and ecdysozoans should permit the deduction of the role of the gene in the ancestor of the eubilaterians (the PDA). Such comparisons of Gsx expression, however, present a conundrum; was ancestral Gsx expression simple or complex, with subsequent extensive secondary modifications required in both scenarios? Detailed similarities between *Platynereis *and vertebrates lead us to favour the hypothesis that the ancestor (in terms of its Gsx expression pattern) was complex, with Gsx domains in a variety of nervous system roles (eyes, neurosecretory cells and 'hindbrain', and potentially along the anterior-posterior axis of the nerve cord which was not examined here) (Figure 4). Gsx expression was then secondarily simplified in several lineages. This secondary simplification led to a similar outcome in each of the simplified cases so far characterised; small patches of expression in the anterior CNS in the polychaete *Capitella *[7], the chordates amphioxus [1,10] and *C. intestinalis *[30], and possibly the sea urchin [29]. The alternative, of an ancestor with simple Gsx expression (perhaps restricted to a small area in the anterior CNS), would then require independent elaboration of the expression in the lineages leading to *Platynereis *and vertebrates. There is no reason to suppose that such independent elaboration would necessarily follow similar avenues in multiple cell types and tissues in different lineages. Indeed it seems unlikely that this should be the case, and so the extensive detailed similarities between the Gsx expression of *Platynereis *and vertebrates can be taken as indicating a complex pattern of Gsx expression in the PDA, with roles in eyes, neurosecretory cells and regionalisation of the neural tube/column.

In addition to the neural expression of *Pdu-Gsx*, there is expression in the developing mouth, the stomodeum, and then later in the midgut (Figure 4). The expression of *Pdu-Gsx *in the stomodeum is intriguing in the context of the hypothesis that the ParaHox genes patterned the gut tube of the PDA, and the anterior gut-patterning role of Gsx was lost on the deuterostome lineage with the evolution of the secondary mouth [63,64]. Gsx expression in a protostome stomodeum has only been documented in another nereid polychaete, *N. virens*, prior to this work [38]. Together the *Nvi-Gsh *and the *Pdu-Gsx *expression described here may lend support to the ancestral mouth patterning role of Gsx (and the lack of complete homology, at the level of developmental patterning, of the mouths of protostomes and deuterostomes [65]). Clearly a wider range of protostomes must be sampled to obtain a consensus on the role of Gsx in the stomodeum of these animals.

The expression of *Pdu-Xlox *during midgut development is similar to the Xlox expression in other annelids [7,38-40]. Since deuterostome Xlox genes are also expressed in midgut regions during embryogenesis, it is likely that a role for Xlox in midgut development was present in the PDA. *Pdu-Xlox *is also expressed in the nervous system. Neural expression is also known for chordates [1,10,23,31]. An ancient neural role for Xlox in the PDA thus also seems likely in addition to the gut function. If this hypothesis is true, however, it would once again necessitate secondary simplification and loss of neural Xlox expression in some lineages such as *Capitella *[7].

The midgut expression of *Pdu-Xlox *appears very similar to the midgut expression of *Pdu-Gsx *at 6 dpf, and both genes are also expressed in the ventral neural plate in similar locations at the same time (48 to 50 hpf) (Figure 4). Given the fact that the clustering of *Pdu-Gsx *and *Pdu-Xlox *has been conserved since the time of the PDA, perhaps the commonalities in aspects of their expression reflect a shared regulatory mechanism, such as a shared enhancer(s), which in turn constrains this clustered arrangement.

*Pdu-Cdx *has a complex, dynamic expression pattern in cells of the ectoderm, endoderm and possibly the nascent larval mesoderm [54]. This is also the case for Cdx expression in other annelids (*Capitella *[7] and *Tubifex *[55]) and the mollusc *Patella vulgata *[53] in which the expression extends to extremely anterior regions of the embryo. This anterior expression of Cdx in these lophotrochozoans conflicts with what might be expected if the ParaHox genes exhibited spatial colinearity at the time of initial gene activation (Gsx – anterior, Xlox – central, Cdx – posterior). The genomic organisation of the genes is thus a prime consideration, and intriguingly *Pdu-Cdx *has broken away from the ParaHox cluster (Figure 3). Such an arrangement, of Gsx and Xlox being clustered and Cdx separated away, is also present in the mollusc *Lottia gigantea *[66], which may well reflect the situation in *Patella*, although this needs to be specifically tested.

The ParaHox cluster has degenerated still further in the polychaete *Capitella*, in which Gsx has separated from Cdx and Xlox, and there are a couple of non-homeobox genes that have invaded the region between Cdx and Xlox [67] (summarised in Figure 6). *Platynereis *and limpets may then represent the general lophotrochozoan condition for the ParaHox cluster, of Gsx and Xlox clustered and Cdx broken away, which correlates with the relatively anterior initial expression of Cdx up to regions around the mouth and prototroch (Figure 4) [53,54]. Potentially this unusual expression of Cdx has been able to evolve due to this separation of the gene from the rest of the ParaHox cluster, and escape from any pan-cluster regulatory mechanisms analogous to those operating in the vertebrate Hox clusters [60]. The situation has then been taken to further levels of derivation in *Capitella *(and possibly *Tubifex*) with continued ParaHox cluster degeneration. An alternative hypothesis would be that the ancestral expression of Cdx covered an extensive anterior-posterior portion of the nervous system, as it does in the lophotrochozoans described here and also, intriguingly, in the acoels, a possible basal lineage of Bilateria and hence potentially informative PDA outgroup [68]. Since the phylogenetic position of acoels remains uncertain [69,70] and the genomic organisation of the ParaHox genes is not yet known in acoels, their suitability as a PDA outgroup for the Cdx question (is Cdx ancestrally in an intact cluster with posterior expression or with extensive anterior-posterior expression?) is not yet clear.

This hypothesised break-up of the ParaHox cluster by the 'escape' of Cdx in these lophotrochozoans contrasts with alternative possible scenarios in which the ParaHox genes are progressively assembled into a cluster during evolution, and the three-gene cluster of Gsx, Xlox and Cdx was a chordate innovation. Here we favour the first scenario above, of a Gsx-Xlox-Cdx cluster in the PDA followed by a break-up in the protostomes examined, since this is much more parsimonious than the alternative of genes originating by tandem duplication and so being ancestral neighbours (as the ParaHox genes are presumed to have done due to their sequence similarities), followed by separation before the origin of the PDA, and then a secondary re-association in the chordate lineage.

In the context of colinearity we find it intriguing that aspects of spatial colinearity can still be distinguished in the CNS and gut whilst temporal colinearity is absent (notwithstanding the complications of *Pdu-Cdx *expression described above and in [54] which mean that strict spatial colinearity at the time of gene initiation is not observed). It has been speculated that pan-cluster mechanisms responsible for temporal colinearity in Hox clusters act as the principal constraining force maintaining those clusters, whilst spatial colinearity can remain even after the clusters are broken up [2,35,71,72]. Here we show that the evolutionary sister to the Hox cluster, the ParaHox cluster, may conform to the same paradigm, with *Pdu-Gsx *initiated in the anterior CNS and gut (stomodeum), *Pdu-Xlox *in the ventral plate and midgut, and *Pdu-Cdx *resolving to the posterior. However, in terms of the timing of initiation the posterior gene (*Pdu-Cdx*) and anterior gene (*Pdu-Gsx*) are both activated before the middle gene (*Pdu-Xlox*).

### The ParaHox genomic neighbourhood in the protostome-deuterostome ancestor

Figure 5 shows the reconstruction of the genomic region around the ParaHox cluster of the PDA, which almost certainly contained AIR1L, Btk, Rad50 and CcnA genes, and may well also have contained Exosc9, Lamc1/3 and SF1KHL genes. This level of synteny conservation between *Platynereis *and humans, and the ability to reconstruct the arrangement of genomic regions from an ancestral genome that existed over 550 million years ago, implies a strikingly low level of genomic rearrangement on the *Platynereis *and human lineages, at least in this ParaHox region. Whether such a 'stable' arrangement of the genome extends more widely in *Platynereis*, or instead is something distinctive about the ParaHox region, remains to be seen.

By analogy to vertebrates, both the Hox clusters and the single remaining ParaHox cluster are located in GRBs [61]. Such regions are refractive to genomic rearrangements due to the presence of regulatory elements that are acting over large distances. Whilst the order of the genes around the ParaHox cluster is different between *Platynereis *and humans, and indeed the *Platynereis *cluster has been broken, which indicates that the polychaete ParaHox genes have not been retained in a GRB comparable to that of vertebrates, the striking level of synteny may be indicative of a ParaHox GRB that has only recently been lost in the *Platynereis *lineage. The alternative, that the high level of synteny between humans and *Platynereis *is widespread beyond the ParaHox loci, would provide a further example of the prototypical nature of *Platynereis *biology and its huge potential for revealing the nature of the PDA [73,74].

Further sequencing data around the *Platynereis *ParaHox genes will also be interesting with regards to seeing whether the ParaHox neighbouring genes that have permitted the detection of the ParaHox locus of the cnidarian, *Nematostella vectensis *[5], also neighbour the Platynereis ParaHox locus. The ParaHox neighbours described here for *Platynereis *are not present on the ParaHox scaffold of *Nematostella*. Potentially these neighbours of the PDA ParaHox genes (in Figure 5) only became associated with the ParaHox locus after the separation of the cnidarian and bilaterian lineages.

## Conclusion

This putative prototypical nature of *Platynereis *genome organisation and development within the protostomes leads us to hypothesise that the PDA had a nervous system with a complex pattern of Gsx expression. Also, the expression of *Pdu-Gsx *in stomodeal development may provide molecular evidence for an ancestral role for Gsx in mouth patterning. The PDA is likely to have had a neural Xlox role, in addition to a widely conserved function in midgut development, and the expression of Cdx in lophotrochozoans such as *Platynereis *highlights the importance of considering gene expression in the context of genome organisation. The scope for reconstructing large areas of ancient genomes deep in animal ancestry has recently been greatly enhanced with the discovery that high levels of synteny exist between the genomes of the cnidarian, *Nematostella vectensis *and humans [75]. Distinguishing the changes between this diploblast-triploblast ancestral genome and the ancestral bilaterian genome, which subsequently gave rise to the huge diversity of bilaterian animal life, requires comparisons between deuterostomes such as humans and a prototypical protostome genome. If the synteny around the ParaHox cluster is indicating the prototypical nature of the *Platynereis *genome, then this polychaete may be an excellent candidate for large-scale genomic sequencing and studies of genome organisation.

## Methods

### Cloning and BAC library screening

Partial fragments of *Pdu-Gsx *and *Pdu-Xlox *were isolated with the primers GsxIb, GsxIIb, GsxIIIb XloxIc and SO2, with the conditions described in [6]. A cDNA fragment of *Pdu-Gsx *was then isolated as described in [57] [Genbank: EF384214]. A longer fragment of *Pdu-Xlox *coding sequence [Genbank: FJ001341] was then isolated by 5'RACE PCR from a mixture of *P. dumerilii *libraries of different stages (24 hpf, 48 hpf and 72 hpf) that were prepared using the SMART RACE cDNA amplification kit (Clontech) according to the manufacturer's recommendations. The primers used were xlox_lo1: ATCTTCTGAGTAAGCAGAATCTGGAG (first PCR) and xlox_lo2: CTCTGTCATTCGCGTTCTGTTC (nested PCR), along with the universal primer mix (UPM) provided by the manufacturer. PCR conditions were: 6 cycles of (1 min at 95°C; 2 min at 62°C; 4 min at 68°C); followed by 36 cycles of (1 min at 95°C; 2 min at 60°C; 4 min at 68°C) and a final extension at 72°C for 10 min. The *Pdu-Cdx *cDNA was that from [54], where it was called *Pdu-cad*. *Pdu-Cdx *is used here for consistency and by extension from the chordate ParaHox nomenclature. These fragments were used to screen a *Platynereis *BAC library [74]. Probes were synthesised with the PCR DIG labelling mix kit (Roche) and 25 ng/ml in DIG Easy Hyb solution (Roche) used to screen the library at 42°C, followed by washes in 0.5× SSC/0.1%SDS for 2 × 15 to 30 min at room temperature, and then 0.2 × SSC/0.1% SDS at 65°C for 2 × 40 min. Signals were detected by following the instructions for the DIG nucleic acid detection kit (Roche), but with the anti-digoxigenin-AP used at 1:20,000 and CDP-Star chemiluminescence substrate instead of NBT-BCIP. BAC DNA was purified with the QIAfilter Plasmid Midi kit (Qiagen) and gene content confirmed by PCR. Entire BAC clones were sequenced as described in [74].

### Whole mount in-situ hybridisation

*P. dumerilii *embryos were obtained from breeding cultures, following [76], and were raised at 18°C. Larvae were fixed and subjected to WMISH as previously described [77].

### Fluorescent In-Situ Hybridisation

Chromosome spreads were prepared as described in [78]. The procedures for two-colour FISH were modified from [78] as follows. BAC clones were labelled with digoxigenin or fluorescein-12-dUTP and Nick translation mix (Roche). The hybridisation mix contained both DIG and fluorescein-labelled probes, each at a concentration of 11.1 ng/μl. DIG-labelled probes were detected with anti-DIG-rhodamine Fab fragments (1:200; Roche) for 1 hr, followed by Texas-Red anti-sheep (1:100; Vector Laboratories) for 30 min. Fluorescein-labelled probes were detected with the Alexa Fluor 488 Signal-Amplification kit (Molecular Probes). Slides were washed in 4 × TNFM [78] between each application (three times after the first and the last, and two times for the second, 5 min each). After the last wash, slides were further washed in 4 × SSC, 0.05% Tween20 twice for 5 min at room temperature. Slides were then equilibrated with 1× PBS before mounting with Vectashield antifadent agent containing DAPI (Vector Laboratories) for chromosome counterstaining. Images were captured with a Zeiss Axioskop microscope equipped with an Axiocam camera. All images were processed with whole-layer colour adjustment on the complete image with Adobe Photoshop 7.0.

### Annotation of the BAC sequences and orthologous locations in human

BAC clone sequences containing *Pdu-Gsx *(clone 233N17), and *Pdu-Gsx *and *Pdu-Xlox *(clone 76L3) were assembled into a contig of approximately 220 kb with the bl2seq program at the NCBI. Three BAC clones containing *Pdu-Cdx *(174K23, 219I5, 155I15) were assembled into a contig of approximately 230 kb. BAC sequences described in this work are deposited in the Genbank with accession numbers [Genbank: FJ001337–FJ001340].

To find additional genes in these contigs besides the ParaHox genes, the entire contig sequences were scanned with GENSCAN and FGENESH (implemented at www.softberry.com [79]), and all predicted peptide sequences searched against Genbank with BLASTP. Also, overlapping 10 kb windows of each contig were searched against Genbank with BLASTX. Combining the output from each of these approaches, models were excluded that contained obviously repetitive sequences, low sequence similarities to other bilaterians (less than 20%), or widespread stop codons. Models with similarities to reverse transcriptases or transposases were also excluded. GenomeScan was used to refine the gene models, using the amino acid sequences of proteins isolated by the BLASTP searches above. Predicted amino acid translations of the putative genes were aligned to candidate orthologues from other taxa with ClustalX 1.83. For the ParaHox genes, phylogenetic trees were constructed with Bayesian (amino acid substitution models were sampled in proportion to posterior probability using the model jumping command, 1,000,000 generations, MrBayes-3.1.2), Neighbour-Joining (ClustalX 1.83), and Maximum-Likelihood (JTT model, 1,000 bootstrap replicates, Phyml_v2.4.4) analyses. Neighbour-Joining trees were also constructed for the neighbouring genes (ClustalX 1.83) to help confirm their identity. Genes were named according to the common conserved domains, phylogenetic analysis and the latest nomenclature of respective gene families. The orthologous gene locations in human were located according to the Map Viewer (Human Build 36.2).

## Abbreviations

BAC: bacterial artificial chromosome; CNS: central nervous system; dpf: days post fertilisation; FISH: fluorescent *in situ *hybridisation; GRB: genomic regulatory block; hpf: hours post fertilisation; PDA: protostome-deuterostome ancestor; WMISH: whole mount *in situ *hybridisation

## Authors' contributions

DEKF conceived and supervised the project, and drafted and coordinated the writing of the paper. JHLH and NK isolated the BAC clones. JHLH performed the chromosome FISH. SS, GM, CJ, and BS sequenced the BAC clones. FR, ND, GB and DA assembled the expression data. JHLH and DEKF analysed the sequences. JHLH, FR, GB, DA and DEKF wrote the paper. All authors read and approved the final manuscript.

## Supplementary Material

Additional file 1 **Supplementary sequence characterisation, materials and methods, sequence alignments and phylogenetic trees.** Click here for file
