# Supplementary material for: Features of the ancestral bilaterian inferred from Platynereis dumerilii ParaHox genes
Source: BMC Biol. 2009 Jul 23;7:43. doi: 10.1186/1741-7007-7-43 (PMC2723086; doi:10.1186/1741-7007-7-43)
Supplement: Additional file 1 — Supplementary sequence characterisation, materials and methods, sequence alignments and phylogenetic trees. [file 1741-7007-7-43-S1.doc]

**Supplementary Information.**

Characterization of the *P*. *dumerilii* ParaHox gene neighbours.

The putative Matrilin-like protein (Pdu-MatnL) contains NIDO, von Willebrand factor (vWF) type D, two calcium binding EGF-like domains, and three vWA Matrilin domains as predicted from comparisons to the Conserved Domain Sequences (CDS) at NCBI. Such domain organisation is similar, but not identical, to Matrilin proteins in chordates, which contain only the vWA Matrilin and EGF-like domains [1-4]. Some other proteins have some similarity to this *P*. *dumerilii* sequence, detected by BLAST, but these other proteins (e.g. Fibrillin, Latent-Transforming Growth Factor, and Notch) do not contain the distinctive vWA domain that is present in Matrilins. This *P.dumerilii* sequence possesses a vWA domain that has most similarity to the vWA domains of Matrilin proteins, and hence we name the gene *Pdu-MatnL* (Fig. S5).

Pdu-Lamc1/3 contains a Laminin N-terminal domain (domain VI) and Laminin EGF-like domains (Domains III and V) according to the CDS at NCBI. From amino acid alignments and phylogenetic trees *Pdu-Lamc1/3* can be classified as encoding a Laminin Gamma 1 or Laminin Gamma 3 protein, rather than a Netrin, where these two distantly related families have similar N-terminal domain structures [5, 6] (Fig. S6). Sequence alignments of Pdu-Lamc1/3 to orthologous proteins from other taxa reveal that it is likely that the C-terminus part of the coding sequence of the gene is not within the sequenced contig.

The Pdu-AIR1L amino acid sequence contains a clear AIR1 domain and also a possible ComEA domain, as predicted by the CDS at NCBI (Fig. S7). This similarity to a ComEA domain is however low, and a ComEA and AIR1 combination has not yet been found in any other animal. Pdu-AIR1L has been named on the basis of its possession of an AIR1 domain. This domain is named from the Arginine Methyltransferase-Interacting protein that contains the RING finger domain for posttranslational modification, protein turnover, chaperone function or intracellular trafficking and secretion [7].

The putative *Pdu-Btk* gene encodes a protein with a PH (pleckstrin homology) domain, Zinc-binding motif, SH3 (Src homology 3) domain, SH2 (Src homology 2) domain, and a tyrosine kinase catalytic domain (Fig. S8). This domain structure permits a confident classification of the *Pdu-Btk* in the distinct family of Tyrosine Kinase which includes Bruton's Agammaglobulinemia Tyrosine Kinase (Btk), Tec, Itk, Bmx and Txk, which share the same domain structure [8].

Pdu-SF1KHL is a putative protein containing a Splicing Factor 1 K Homology RNA-binding (SF1-like-KH) domain, and the gene is located around 30kb from the start Methionine codon of *Pdu-Cdx* (Fig. 2). SF1-like-KH domain-containing RNA-binding proteins that are highly similar to this *Platynereis* sequence have been identified in insects and mammals, but have not been named (Fig. S9).

*Pdu-Rad50* is found 16kb away from the start Methionine codon of *Pdu-SF1KHL.* It has high sequence similarity to both Rad50 and Myosin genes. However conserved domain sequence with the catalytic domains of Rad50 along with the predicted protein structure lacking the distinctive head and filament organisation of Myosin proteins, are consistent with this gene being the Rad50 DNA repair gene of *P*. *dumerilii* rather than Myosin (Fig. S10).

*Pdu-Ccna* is located around 18kb away from the start Methionine codon of *Pdu-Rad50*. The amino acid sequence contains both N-terminal and C-terminal cyclin domains. Mitotic Specific Cyclins are essential for the control of the cell cycle at the G2/M (mitosis) transition, and consist of Cyclin A and B subfamilies. Due to a high level of sequence similarity and as suggested by phylogenetic analysis, it is clear that the putative *P*. *dumerilii* Cyclin gene belongs to subfamily A rather than subfamily B (Fig. S11).

*Pdu-Exosc9* is located 32kb away from the start Methionine codon of *Pdu-Ccna*. Similar to other Exoribonuclease family members, RNase PH domains 1 and 2 are found in the sequence [9]. Sequence alignments and phylogenetic analysis reveal greater similarity to Exosome Component 9 than Exosome Component 7 or 8 genes, which have the same domain structure [10] (Fig. S12).

**Supplementary References**

1. Deak F, Wagener R, Kiss I, Paulsson M: **The matrilins: a novel family of oligomeric extracellular matrix proteins**. *Matrix Biol* 1999, **18**(1):55-64.

2. Whittaker CA, Hynes RO: **Distribution and evolution of von Willebrand/integrin A domains: widely dispersed domains with roles in cell adhesion and elsewhere**. *Mol Biol Cell* 2002, **13**(10):3369-3387.

3. Ko YP, Kobbe B, Paulsson M, Wagener R: **Zebrafish (Danio rerio) matrilins: shared and divergent characteristics with their mammalian counterparts**. *Biochem J* 2005, **386**(Pt 2):367-379.

4. Wagener R, Ehlen HW, Ko YP, Kobbe B, Mann HH, Sengle G, Paulsson M: **The matrilins--adaptor proteins in the extracellular matrix**. *FEBS Lett* 2005, **579**(15):3323-3329.

5. Hohenester E, Engel J: **Domain structure and organisation in extracellular matrix proteins**. *Matrix Biol* 2002, **21**:115-128.

6. Yurchenco PD, Wadsworth WG: **Assembly and tissue functions of early embryonic laminins and netrins**. *Curr Opin Cell Biol* 2004, **16**(5):572-579.

7. Inoue K, Mizuno T, Wada K, Hagiwara M: **Novel RING finger proteins, Air1p and Air2p, interact with Hmt1p and inhibit the arginine methylation of Npl3p**. *J Biol Chem* 2000, **275**(42):32793-32799.

8. Vihinen M, Mattsson PT, Smith CI: **Bruton tyrosine kinase (BTK) in X-linked agammaglobulinemia (XLA)**. *Front Biosci* 2000, **5**:D917-928.

9. Houseley J, LaCava J, Tollervey D: **RNA-quality control by the exosome**. *Nat Rev Mol Cell Biol* 2006, **7**(7):529-539.

10. Chen CY, Gherzi R, Ong SE, Chan EL, Raijmakers R, Pruijn GJ, Stoecklin G, Moroni C, Mann M, Karin M: **AU binding proteins recruit the exosome to degrade ARE-containing mRNAs**. *Cell* 2001, **107**(4):451-464.

11. Ferrier DEK, Dewar K, Cook A, Chang JL, Hill-Force A, Amemiya C: **The chordate ParaHox cluster**. *Curr Biol* 2005, **15**(20):R820-822.

12. de Rosa R, Prud'homme B, Balavoine G: ***Caudal* and *even-skipped* in the annelid *Platynereis dumerilii* and the ancestry of posterior growth**. *Evol Dev* 2005, **7**(6):574-587.

13. Arnone MI, Rizzo F, Annunciata R, Cameron RA, Peterson KJ, Martinez P: **Genetic organization and embryonic expression of the ParaHox genes in the sea urchin S. purpuratus: insights into the relationship between clustering and colinearity**. *Dev Biol* 2006, **300**(1):63-73.

**Supplementary Materials and Methods**

All analyses were carried out using amino acid sequences. Sequences were aligned using the program ClustalX v.2.0.10, with subsequent editing carried out by eye. For the ParaHox genes, phylogenetic relationships were estimated by Neighbour-Joining, Maximum Likelihood and Bayesian Inference, with phylogenetic support (N-J bootstrap support values/ M-L bootstrap support values/ clade posterior probabilities) displayed on the clade credibility tree recovered by Bayesian Inference. Bayesian analyses were carried out in MRBAYES v3.1.2, using the (MC)3 algorithm, with four simultaneous Markov chains per run (three heated, one cold) and two independent runs per analysis as implemented by default. As opposed to a priori selection of a specific amino acid substitution model, the model jumping command in MrBayes was implemented, which selects substitution models in proportion to their posterior probability. Chains were run for 1 million generations with a sampling frequency of 100 generations, resulting in 10,000 samples per run. After chain completion, a plot of log-likelihood against sample number was examined manually for each run in order to judge if stationarity had been achieved and to determine the burn-in. Bayesian posterior probabilities were estimated for each clade from the 50% majority-rule consensus tree of the sampled trees minus the burn-in. Neighbour joining analyses were done with ClustalX v.2.0.10. Maximum likelihood analyses were carried out using PhyML v2.4.4, with the substitution models selected by the Akaike information criterion as implemented in Prottest 1.4. The model selected was JTT. For other neighbour genes, phylogenetic relationships were estimated by Neighbour Joining and Maximum Likelihood with phylogenetic support (N-J bootstrap support values/ M-L bootstrap support values) displayed on the tree recovered by Neighbour Joining. Methods were the same as described above except the models selected for maximum likelihood analyses for Lamc, Ccna, and Exosc datasets were WAG+I+G+F, JTT+I+G+F and RtREV+I+G+F, respectively.

**Supplementary Figure Legends.**

Fig. S1. ParaHox gene phylogeny. Rooted Bayesian phylogenetic tree (amino acid substitution models were sampled in proportion to posterior probability using the model jumping command, 1,000,000 generations, MrBayes-3.1.2). The tree is built with the amino-acid sequences of the homeodomain. Only bootstraps above 0.50 are shown. Groupings of the ParaHox genes are strongly supported. Abbreviations: AmphiCdx, AmphiGsx, AmphiHox1, AmphiHox2, AmphiHox3, AmphiHox4, AmphiHox5, AmphiHox6, AmphiHox7, AmphiHox8, AmphiHox9, AmphiTlx, AmphiXlox, cephalochordate *Branchiostoma floridae* (Cdx, NM_001078201)(Gsx, [11])(Hox1, Z35142)(Hox2, Z35143)(Hox3, P50901)(Hox4, Z35144)(Hox5, Z35145)(Hox6, Z35146)(Hox7, Z35147)(Hox8, Z35148)(Hox9, Z35149)(Tlx, CAD83853)(Xlox, [11]); CinIPF1, CinGsx, urochordate *Ciona intestinalis* (IPF1, NM_001032501)(Gsx, NM_001032491); CspCdx, CspGsx, CspXlox, polychaete *Capitella sp*. *I* (Cdx, DQ102389)(Gsx, AAZ23124)(Xlox, DQ102390); DmeInd, DmePb, DmeCaudal, DmeVnd, arthropod *Drosophila melanogaster* (ind, AAC97116)(proboscipedia, P31264)(caudal, AAF53923)(vnd, X87141); DreGsh1, DreGsh2, zebrafish *Danio rerio* (Gsh1, NP_001012251)(Gsh2, NP_001020683); EscAntp, EscLox4, mollusc *Euprymna scolope* (Antp, AAL25809) (Lox4, AAL25810); MmuCdx1, MmuCdx2 and MmuCdx4, MmuGsh1 and MmuGsh2, MmuIpf1, vertebrate *Mus musculus* (Cdx1, AAH19986) (Cdx2, NP_031669) (Cdx4, NP_031700)(Gsh1, NM_008178)(Gsh2, NM_133256) (IPF1, CAA52389); NviHox3, NviDfd, NviLab, NviLox2, NviLox5, NviPb, NviPost1, NviPost2, NviScr, polychaete *Nereis virens* (Hox3, AAD46168) (Dfd, AAD46169) (Lab, AAD46166)(Lox2, AAD46171)(Lox5, AAD46174) (Pb, AAD46167)(Post1, AAD46175)(Post2, AAD46176) (Scr, AAD46170); PduCdx, PduGsx, PduXlox, PduNK2.1, polychaete *Platynereis dumerilii* (Cdx, [12])(Gsx, this study) (NK2.1, AM114784) (Xlox this study); SpuCdx, SpuGsx, Spulox, sea urchin *Strongylocentrotus purpuratus* (Cdx and Gsx, [13])(lox, AF541970); TcaAbdA, TcaAbdB, TcaAntp, TcaDfd, TcaFtz, TcaInd, TcaLab, TcaScr, TcaUbx, TcaZen, beetle *Tribolium castaneum* (AbdA, NP_001034518)(AbdB, AF227923)(Antp, AAK96031)(Dfd, AAK16423)(Ftz, AAK16421)(Ind, AAW21974)(Lab, AAK96034)(Scr, AAK16422)(Ubx, NP_001034497)(Zen, AAK16424); XlaXLHBOX8, vertebrate *Xenopus laevis* XLHBOX8 (CAA34746).

Fig. S2. Amino acid sequence of Pdu-Gsx aligned to Gsx orthologues from selected bilaterian taxa. Identity with the Pdu-Gsx sequence is highlighted. In addition to the largest block of conservation (homeodomain), there is a region of conservation at the N-terminus of the proteins, the SNAG motif (underlined). Abbreviations: PduGsx, polychaete *Platynereis dumerilii*; PflGsx, hemichordate *Ptychodera flava* (AY436761); PexGsx, clitellate annelid *Perionyx excavatus* (AY769112); MmuGsh1 and MmuGsh2, vertebrate *Mus musculus* (NM_008178 and NM_133256); XtrGsh1 and XtrGsh2, vertebrate *Xenopus tropicalis* (DQ195530 and DQ195531); DmeInd, arthropod *Drosophila melanogaster* (AAC97116). AmphiGsx, cephalochordate *Branchiostoma floridae* [11]; Cin, urochordate *Ciona intestinalis* (NM_001032491).

Fig. S3. Amino acid sequence of Pdu-Xlox aligned with Xlox orthologues from selected bilaterian taxa. Residues identical to Pdu-Xlox are highlighted. In addition to the largest block of conservation (homeodomain), there is a region of conservation just upstream of the homeodomain, the hexapeptide motif (HFPWMK) (underlined). Abbreviations: PduXlox, polychaete *Platynereis dumerilii*; CspXlox, polychaete *Capitella sp*. *I* (DQ102390); Spulox, *Strongylocentrotus purpuratus* (AF541970); XlaXLHBOX8, vertebrate *Xenopus laevis* (CAA34746); MmuIpf1, vertebrate *Mus musculus IPF1* (CAA52389); AmphiXlox, cephalochordate *Branchiostoma floridae* [11].

Fig. S4. Amino acid sequence of Pdu-Cdx aligned with Cdx orthologues from selected taxa. Residues identical to Pdu-Cdx are highlighted. In addition to the largest block of conservation (homeodomain), there is a hexapeptide motif (PYDWMK) just upstream of the homeodomain (underlined). Abbreviations: PduCdx, polychaete *Platynereis dumerilii*; DmeCaudal, arthropod *Drosophila melanogaster* (AAF53923); MmuCdx1, MmuCdx2 and MmuCdx4, vertebrate *Mus musculus* (AAH19986, NP_031669 and NP_031700); AmphiCdx, cephalochordate *Branchiostoma floridae* [11]; Csp, polychaete *Capitella sp*. *I* (DQ102389); PvuCdx, mollusc *Patella vulgata* (AJ518062).

Fig. S5. *Pdu-Matrilin-Like* classification. (a) Translation of Pdu-MatnL, highlighting the distinctive domains (NIDO, vWD, vWA matrilin and EGF-CA).

Fig. S6. *Pdu-Laminin-gamma1/3* classification. (a) Translation of the N-terminal portion of Pdu-Lamc1/3 present in the *Pdu-Gsx/Pdu-Xlox* contig and alignment to the entire amino acid sequences of selected Lamc proteins from other taxa. Residues identical to the Pdu-Lamc1/3 residue are highlighted. The two large blocks of conservation are Laminin N-terminal domain (domain VI) (beneath the rectangular bar) and Laminin EGF-like domains (Domains III and V) (underlined). Abbreviations: Pdu, polychaete *Platynereis dumerilii*; Hsa, human *Homo sapiens* (NM_002293); Ptr, chimpanzee *Pan troglodytes* (XP_001162648); Cfa, dog *Canis familiaris* (XP_537156); Rno, rat *Rattus norvegicus* (XP_341134); Mmu, mouse *Mus musculus* (NP_034813); Dre, zebrafish *Danio rerio* (NP_775384); Aga, mosquito Anopheles gambiae (CAB66001); Aae, diptera *Aedes aegypti* (EAT43380); Dme, fruit fly *Drosophila melanogaster* (NP_524006). (b) Phylogenetic tree of the N-terminal portion of PduLamc1/3 and the N-termini sequences of other selected Lamc proteins and the similar Netrin proteins. Abbreviations: PduLamc, *Platynereis dumerilii*; PtrLAMC1, *Pan troglodytes* laminin gamma 1 (XP_001162648); HsaLAMC1, HsaLAMC2, HsaLAMC3, *Homo sapiens* laminin gamma 1, 2, 3 (CAH70981; NM_005562; ); RnoLamc1, RnoLamc2, RnoLamc3, *Rattus norvegicus* laminin gamma 1, 2, 3 (XP_341134; EDM09550; NP_001101300); MmuLamc1, MmuLamc2, MmuLamc3, *Mus musculus* laminin gamma 1, 2, 3 (NP_034813; NP_032511; CAM26482); AgaLanb2, *Anopheles gambiae* laminin gamma 1 (CAB66001); AaeAAEL005187, *Aedes aegypti* laminin gamma 1 (EAT43380); DmeLanB2, *Drosophila melanogaster* laminin gamma 1 (NP_524006); Amphinetrin, *Branchiostoma floridae* netrin (CAB72422); Gganetrin-1, *Gallus gallus* netrin (NP_990750); MmuNtn1, *Mus musculus* netrin (NP_032770); HumanNTN1, *Homo sapiens* netrin (NP_004813); HmeLNET, *Hirudo medicinalis* netrin (AAC83376); AaeNetrin, *Aedes aegypti* netrin (EAT45717); DmeNetrin-A, DmeNetrin-B, *Drosophila melanogaster* netrin A and B (AAB17533, AAB17534).

Fig. S7. *Pdu-AIR1-Like* classification. (a) Translation of the predicted Pdu-AIR1L gene, with the AIR1 domain underlined. (b) Alignment of the AIR1 domain sequences from the genomes of human, mouse, *Drosophila melanogaster* and *Apis mellifera*, with that of Pdu-AIR1L. Abbreviations: PduAIR1L *Platynereis dumerilii*; Hsa, *Homo sapiens* proteins contain AIR1 domains, HsaZCCHC13, HsaCNBP/ZCCHC22, HsaZCCHC3, HsaZCCHC7, HsaLIN28, HsaZCCHC9, HsahCG_2010686, HsaNLRP1 (NP_976048, NP_003409, CAB81631, NP_115602, NP_078950, NP_115656, EAW595582, EAW90324); Mmu, *Mus musculus* proteins contain AIR1 domains, MmuCnbp, MmuMc14cnbp, MmuZcchc3, MmuLOC100043516, MmuLOC100043390, MmuZcchc9, MmuZcchc7, MmuLin28, MmuENSMUSG00000072994, MmumCG_2332 (NP_038521, CAA77897, EDL05966, XP_001480398, XP_001479659, NP_663428, NP_796001, NP_665832, XP_001472929, EDL02418); Dme, *Drosophila melanogaster* proteins contain AIR1 domains, DmeCG3800, DmeCG9715, DmeGag (AAN16117, NP_648926, CAD65868); Ame, honeybee *Apis mellifera* proteins contain AIR1 domains, AmeLOC725076, AmeLOC725656, AmeLOC413176 (XP_001119951, XP_001121478, XP_396627, XP_394596). Phylogenetic analyses do not resolve whether the *Platynereis* gene is orthologous to any particular AIR1 encoding gene from another animal (data not shown).

Fig. S8. Translation of *Pdu-Btk* and its alignment with other Btk amino acid sequences, highlighting the different putative domains. Residues identical to the Pdu-Btk residue are highlighted in grey. The large blocks of conservation are Pleckstrin homology-like domain (beneath the lines), Bruton's tyrosine kinase Cys-rich motif (inside the rectangle), Src homology 3 domain (inside the black box), Src homology 2 domain (beneath the rectangular bars), and the catalytic domain of tyrosine kinase (underlined). Abbreviations: PduBtk, polychaete *Platynereis dumerilii*; HsaITK, HsaTEC, HsaBTK, HsaTEC, HsaTXK, human *Homo sapiens* (NP_005537, NP_003206, NP_000052, NP_001712, NP_003319); DmeBtk29A, fruit fly *Drosophila melanogaster* (NP_476745); AmeLOC410649, honey bee *Apis* *mellifera* (XP_394126).

Fig. S9. *Pdu-SF1KHL* classification. Amino acid sequence of Pdu-SF1KHL, with the Splicing factor 1 (SF1) K homology RNA-binding domain highlighted, aligned to SF1KH domain-containing proteins from other selected taxa. Residues identical to the Pdu-SF1KHL residue are highlighted. Abbreviations: PduSF1KHL, polychaete *Platynereis dumerilii*; Rno RGD1565775, rat *Rattus norvegicus* (XP_342278); Mmu2810403A07Rik, mouse *Mus musculus* (NP_083090); HsaKIAA0907, human *Homo sapiens* (NP_055764); AmeLOC411122, honeybee *Apis mellifera* (XP_394596); Aae AAEL002621, diptera *Aedes aegypti* (EAT46180).

Fig.S10 *Pdu-Rad50* translation aligned to Rad50 orthologs from other selected taxa. Amino acid identities to the *Platynereis* sequence are highlighted. Abbreviations: Pdu, polychaete *Platynereis dumerilii*; Rno, rat *Rattus norvegicus* (NP_071582); Mmu, mouse *Mus musculus* (NP_033038); HsaRAD50, human *Homo sapiens* (NP_005723); Tca, beetle *Tribolium castaneum* (XP_969783); Dme, fruit fly *Drosophila melanogaster* (NP_726199).

Fig. S11. Pdu-Ccna classification. (a) Amino acid sequence of Pdu-Ccna aligned to other selected cyclin A bilaterian proteins. The main block of conservation is the cyclin domain (underlined). Abbreviations: PduCcna, polychaete *Platynereis dumerilii*; RnoCcna1, RnoCcna2, rat *Rattus norvegicus* (NP_001011949, NP_446154); MmuCcna1, MmuCcna2, mouse *Mus musculus* (NP_031654, NP_033958); HsaCCNA1, HsaCCNA2, human *Homo sapiens* (NP_003905, NP_001228); PvuCcna, gastropod *Patella vulgata* (CAA41254); SsoCcna, bivalve *Spisula solidissima* (CAA38921); TcaLOC661369, beetle *Tribolium castaneum* (XP_972623); DmeCyca, fruit fly *Drosophila melanogaster* (BAA01629). (b) Phylogenetic tree showing the robust classification of the *Platynereis* gene into cyclin A. Other abbreviations: HsaCCNB1, HsaCCNB2, HsaCCNB3, human *Homo sapiens* cyclin B proteins (NP_114172, CAG38558, Q8WWL7); MmuCcnb1, MmuCcnb2, MmuCcnb3, mouse *Mus musculus* cyclin B proteins (AAH80202; CAJ18494; CAM15958); XtrCcnb1, XtrCcnb2, frog *Xenopus tropicalis* cyclin B proteins (AAI58172, CAJ83630); DmeCycb, fruit fly *Drosophila melanogaster* cyclin B protein (CAA07238).

Fig. S12. *Pdu-Exosc9* classification. (a) Amino acid sequence of Pdu-Exosc9 aligned to orthologues from other selected taxa. The RNase PH domains are indicated by underlining. Abbreviations: Pdu, polychaete *Platynereis dumerilii*; HsaEXOSC9, human *Homo sapiens* exosome component 9 (NP_001029366); AmeLOC551855, honey bee *Apis* *mellifera* exosome component 9 (XP_624243); DmeRrp45, fruit fly *Drosophila melanogaster Rrp45* (NP_573163). (b) Phylogenetic tree showing the robust classification of the *Platynereis* gene into subfamily 9 rather than 7 or 8. Abbreviations: PduExosc9, *Platynereis dumerilii*; HsaEXOSC7, human *Homo sapiens* exosome component 7 (NP_055819); HsaEXOSC8, human *Homo sapiens* exosome component 8 (NP_852480); HsaEXOSC9, human *Homo sapiens* exosome component 9 (NP_001029366); AmeLOC551606, *Apis* *mellifera* exosome component 8 (XP_624000); AmeLOC551855, *Apis* *mellifera* exosome component 9 (XP_624243); DmeRrp42, *Drosophila melanogaster Rrp42* (NP_725517); DmeRrp45, fruit fly *Drosophila melanogaster Rrp45* (NP_573163); XlaExosc7, frog *Xenopus laevis* exosome component 7 (NP_001086766); XtrExosc8, frog Xenopus tropicalis exosome component 8 (NP_001007919); RnoExosc7, rat *Rattus norvegicus* exosome component 7 (EDL76765); RnoExosc9, rat *Rattus norvegicus* exosome component 9(AAH97413); BmoExosc7, silkworm *Bombyx mori* exosome component 7 (NP_001040497); DreExosc8, zebrafish *Danio rerio* exosome component 8 (AAI65543); GgaExosc9, chick Gallas gallas exosome component 9 (NP_001030000); MmuExosc7, mouse *Mus musculus* exosome component 7 (NP_001074657); MmuExosc8, mouse *Mus musculus* exosome component 8 (AAH59089); MmuExosc9 mouse *Mus musculus* exosome component 9(AAI58076).


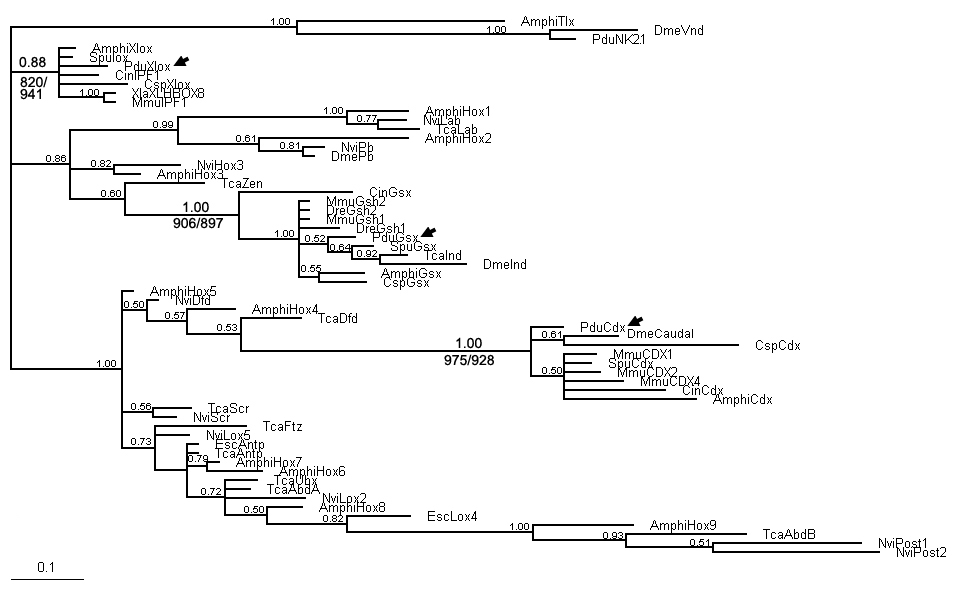


Hui_Fig. S1


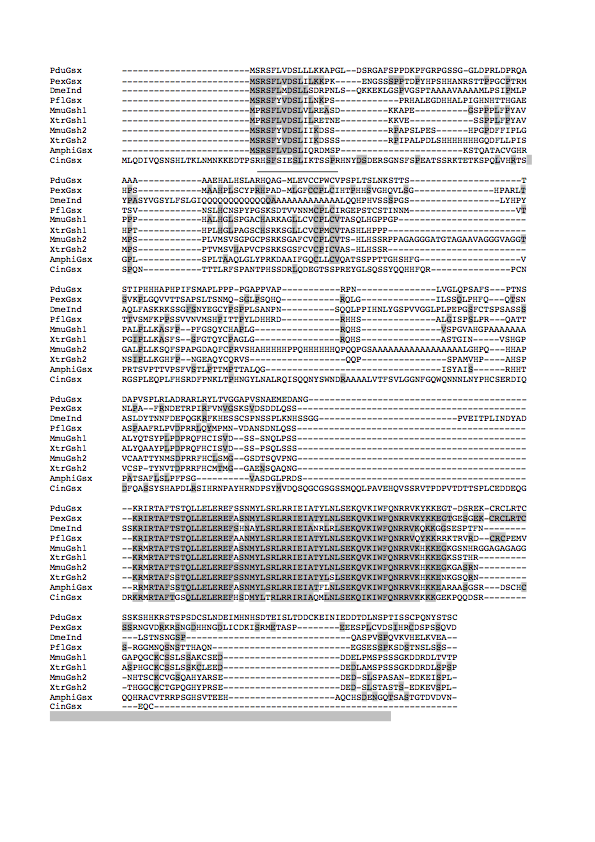


Hui_Fig. S2


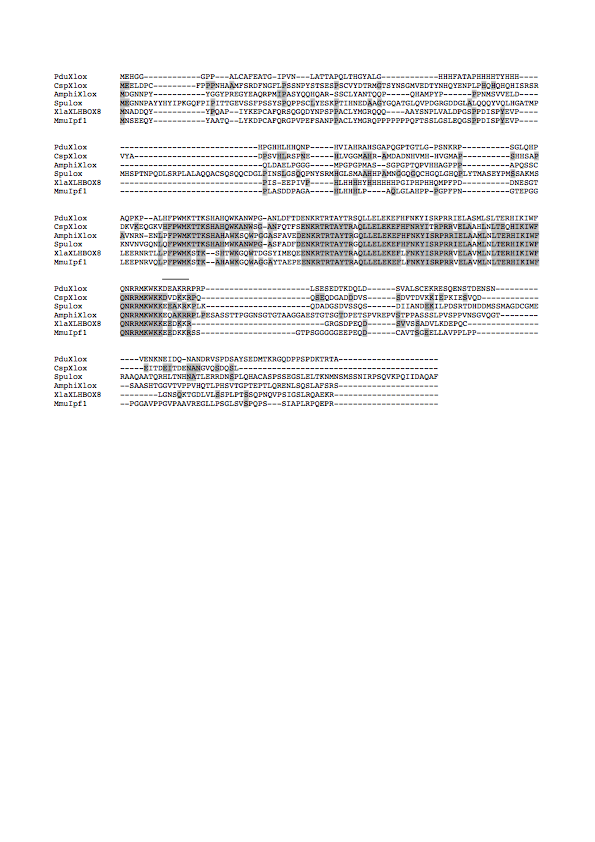


Hui_Fig. S3


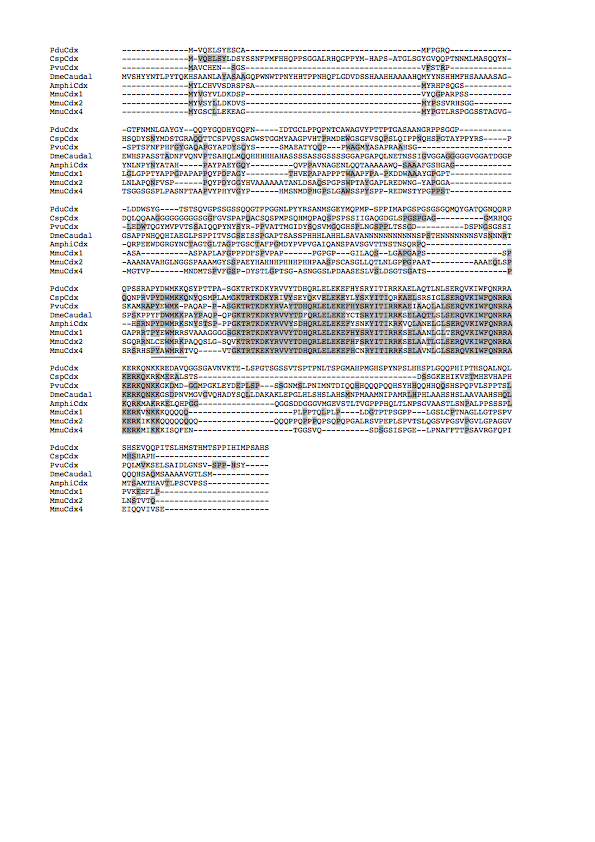


Hui_Fig.S4


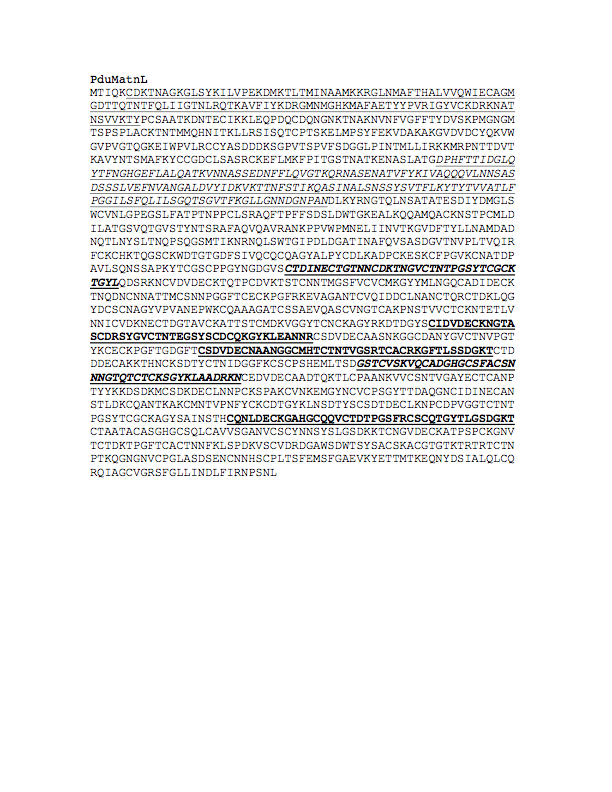


Hui_Fig. S5


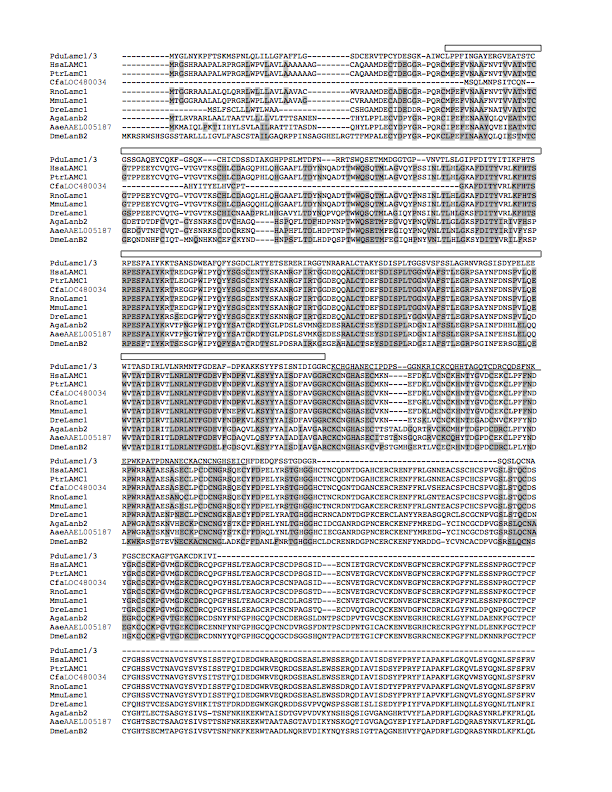


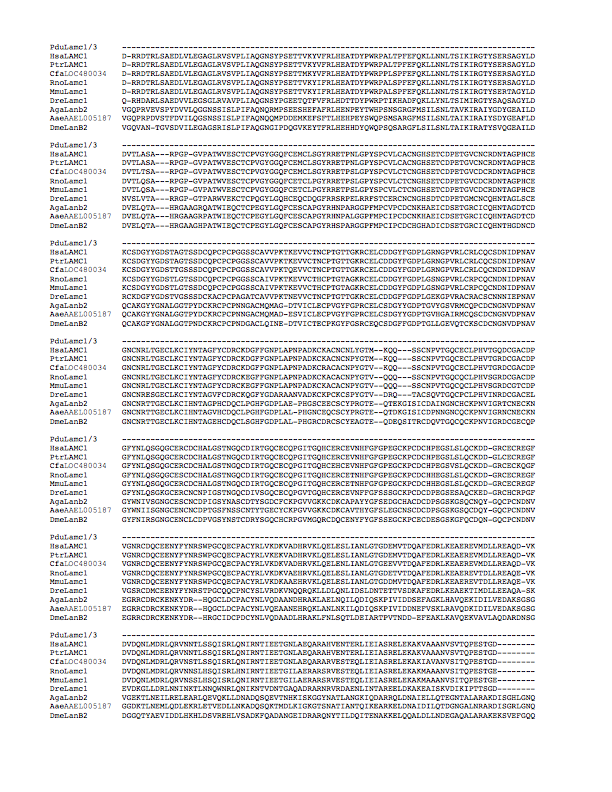


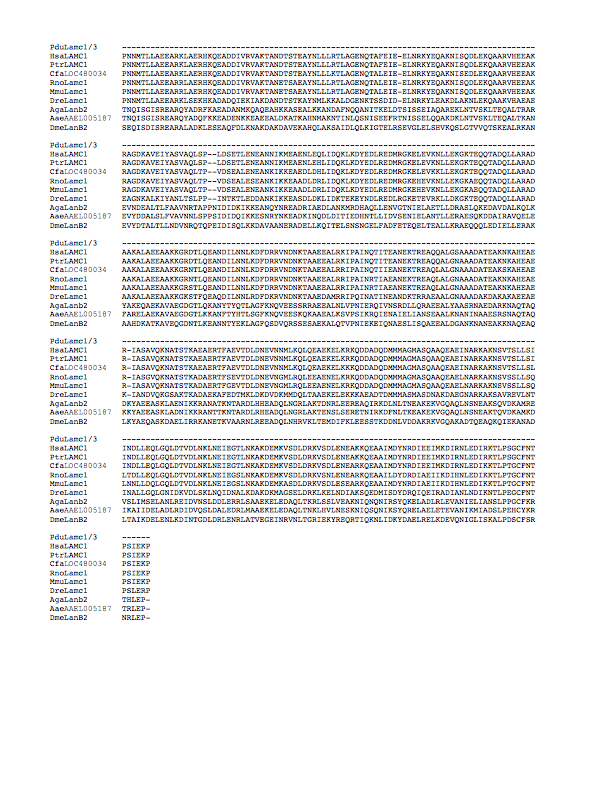


Hui_Fig.S6a


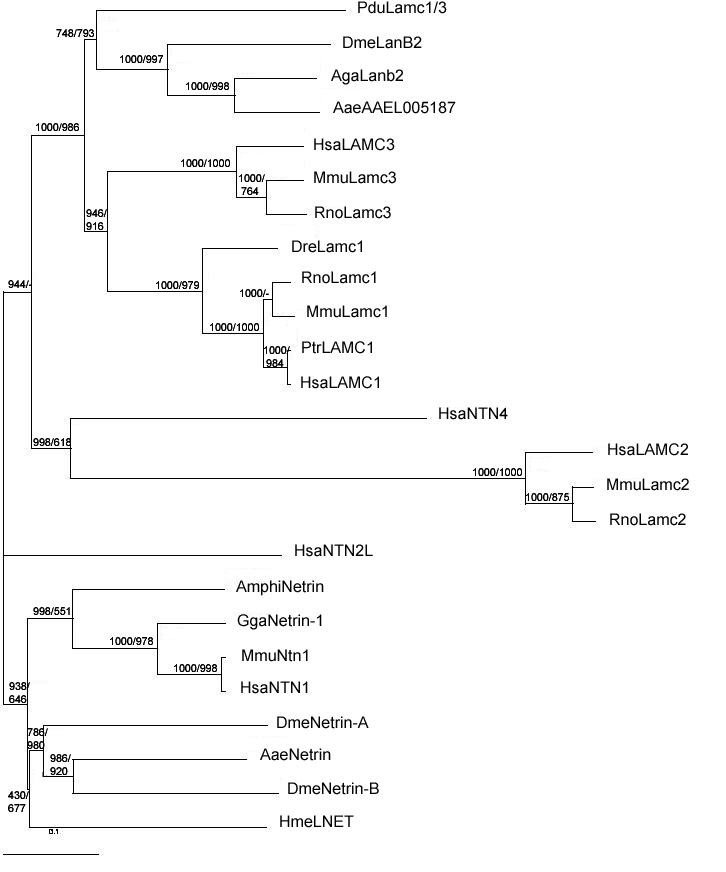


Hui_Fig.S6b

MESKYNINTASKEELMLIEGVNELAAMSIIQYREDVGKITSWYTVKTLAPRTSLDNFMDLHDSGEWSSDIKDFPFRFKIDPPKDNKSTIKSESESSTKLGQANAINGQICNGLHKERPLNSTPSNVLSSLDKDGTDYILMSSSVQKQEVKPAPVPGGSNSSNIDSHYLNLGSKFSNLVYRKLVKNVGRRVNDNSTALCSSSKPHQKSMPKVEKTSSESFQTSTSLSSKKTRTNYSNTAPPSAGNASTDKNFSPVAVNKSVDGVLPPAAEFQFIDGTLTVCLPEAIINGVKRIQLVKPFKSKKEPESSPIPSPVGSPHSAPTPFENLGSGISSSEVPKPRPWKPRTCFECGGAGHLAPHCPTRHQRSIHCFECEGVGHPAPQCSSRRHVSIICHQCRGRGHIAKNCAFSCGPASHFSPRRNITRGYECWNYGHIARNCIDSTSSIVRASNYGSYHHGPSNFNSSLRRLPNQDFKTGSDSQDWRATMRGRI

Hui_Fig. S7a


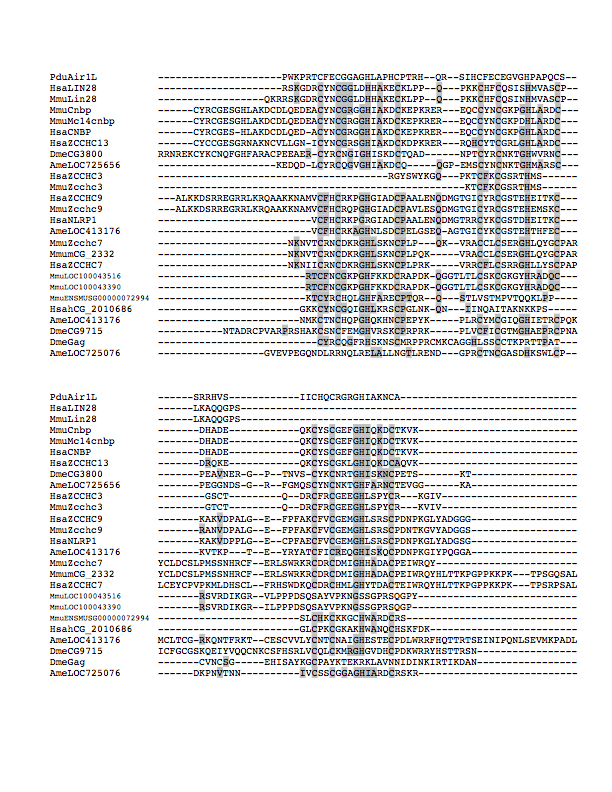


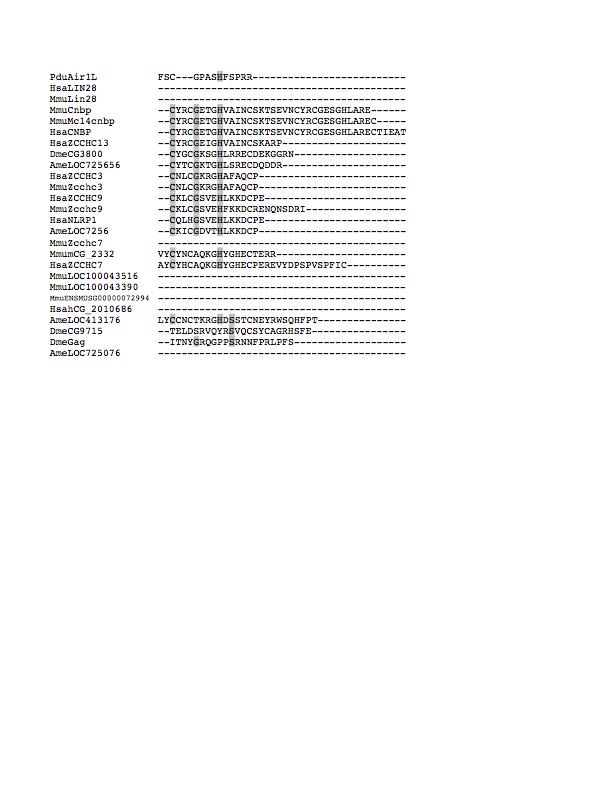


Hui_Fig. S7b


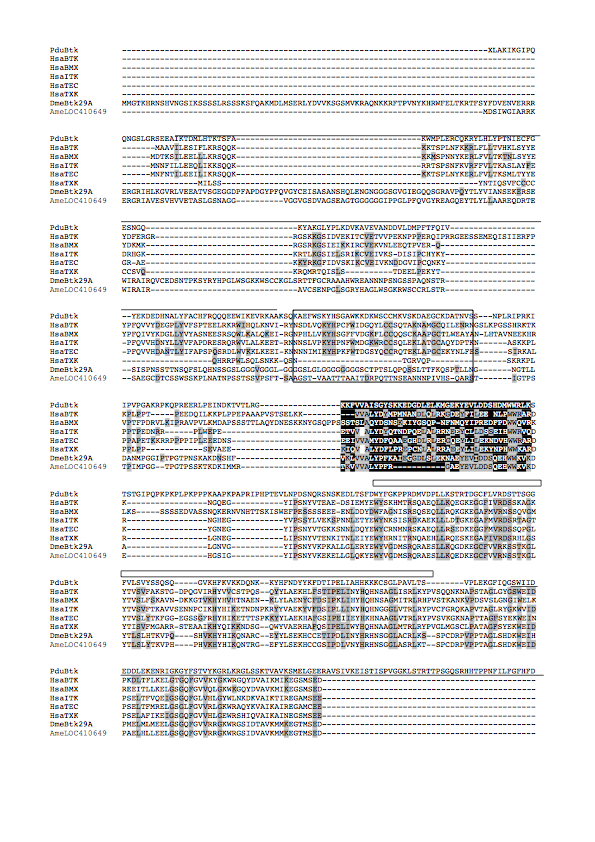


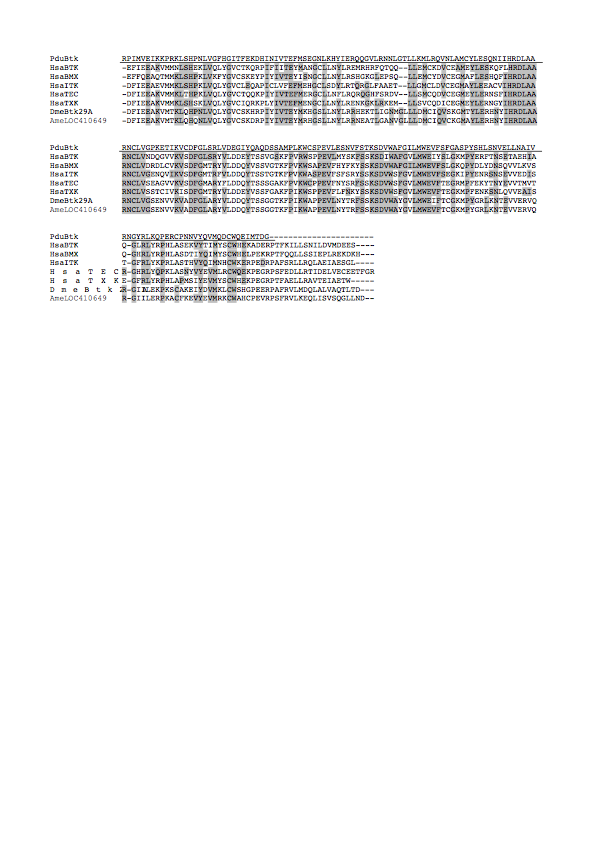


Hui_Fig. S8


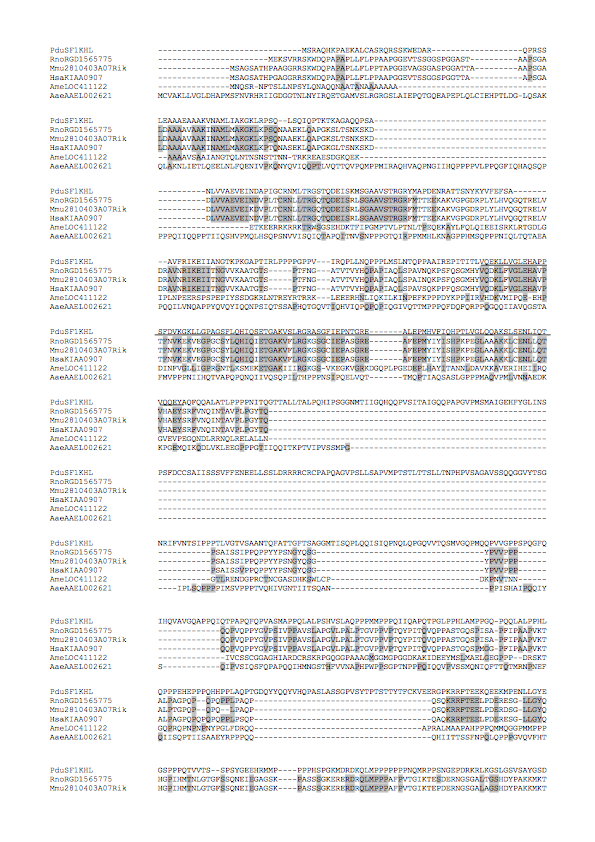


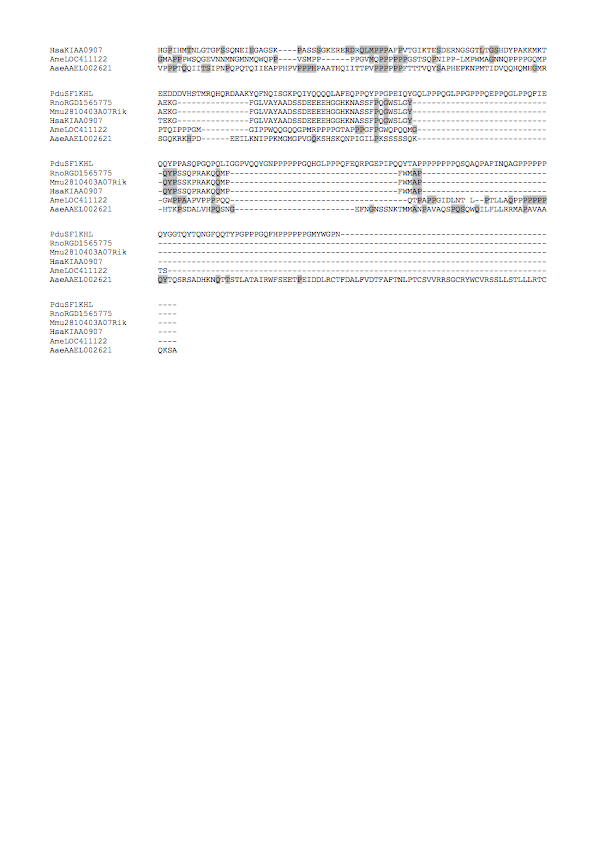


Hui_Fig. S9


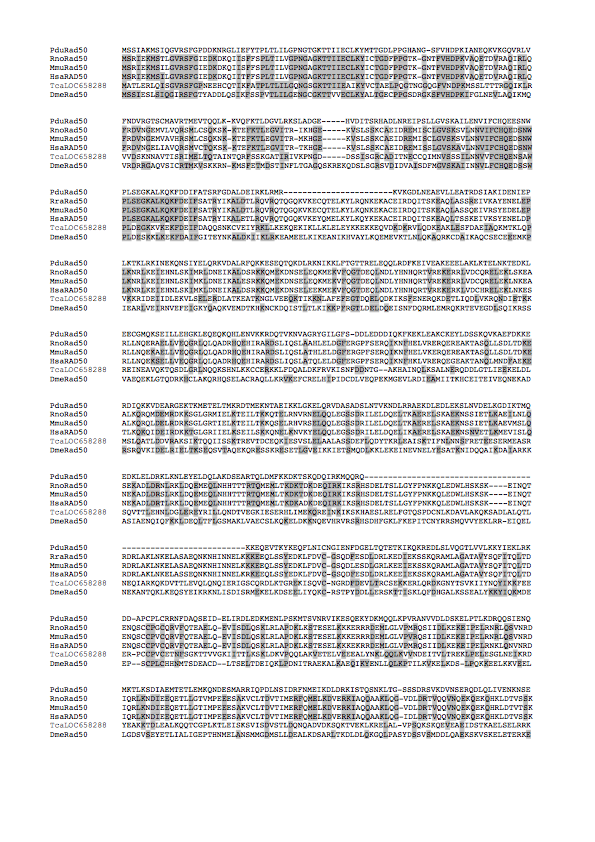


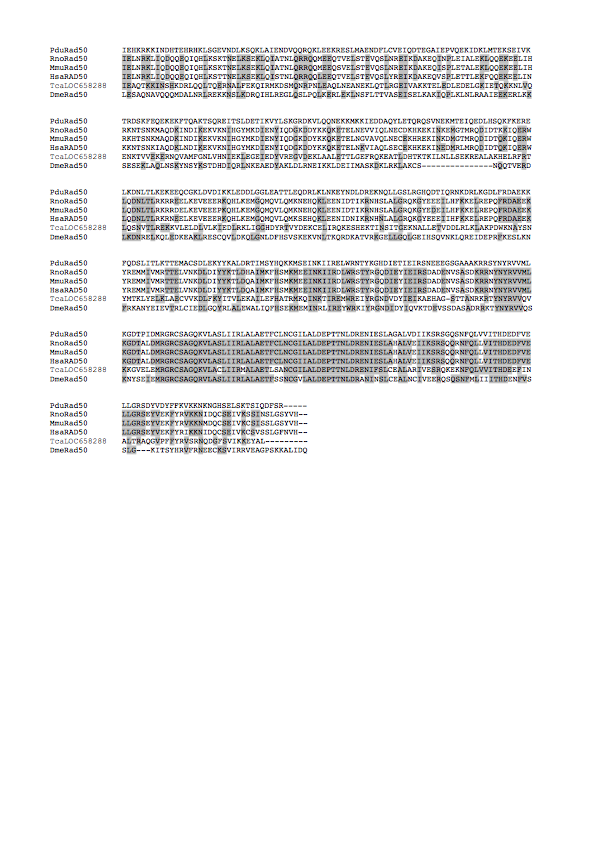


Hui_Fig. S10


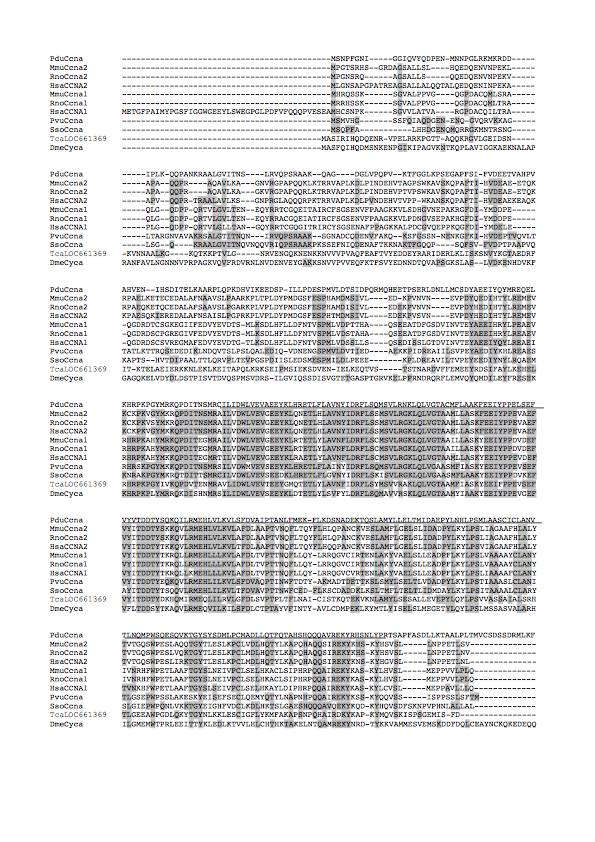


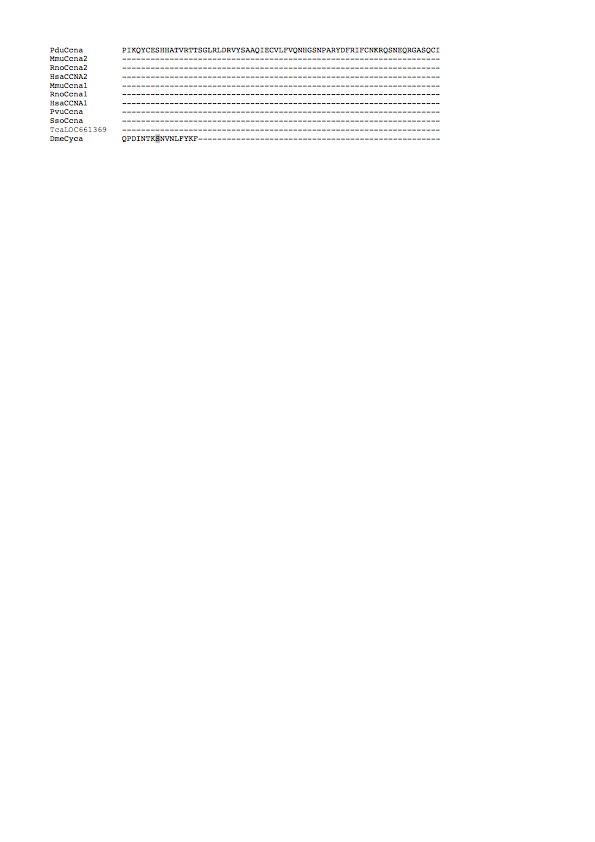


Hui_Fig. S11a


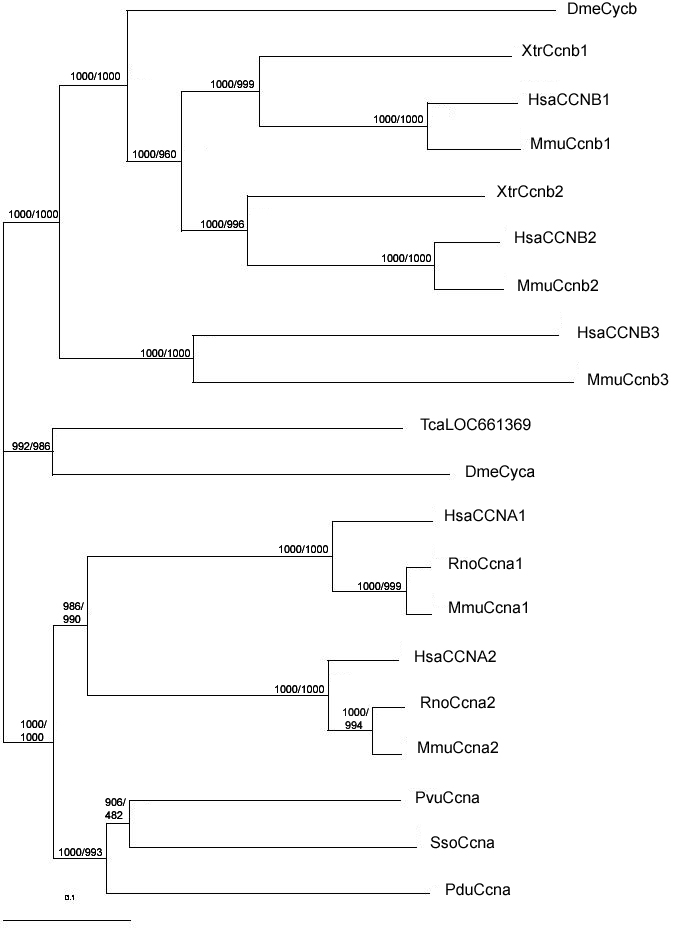


Hui_Fig. S11b


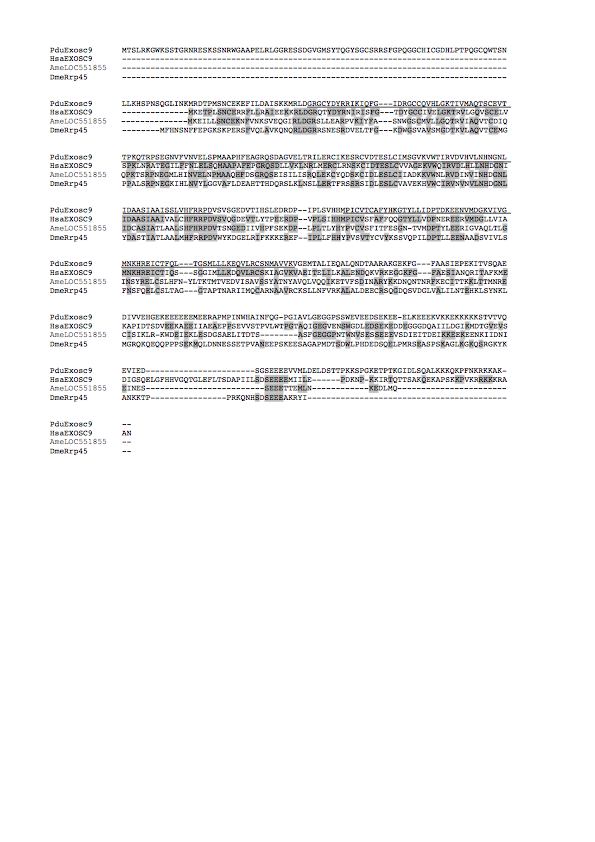


Hui_Fig. S12a


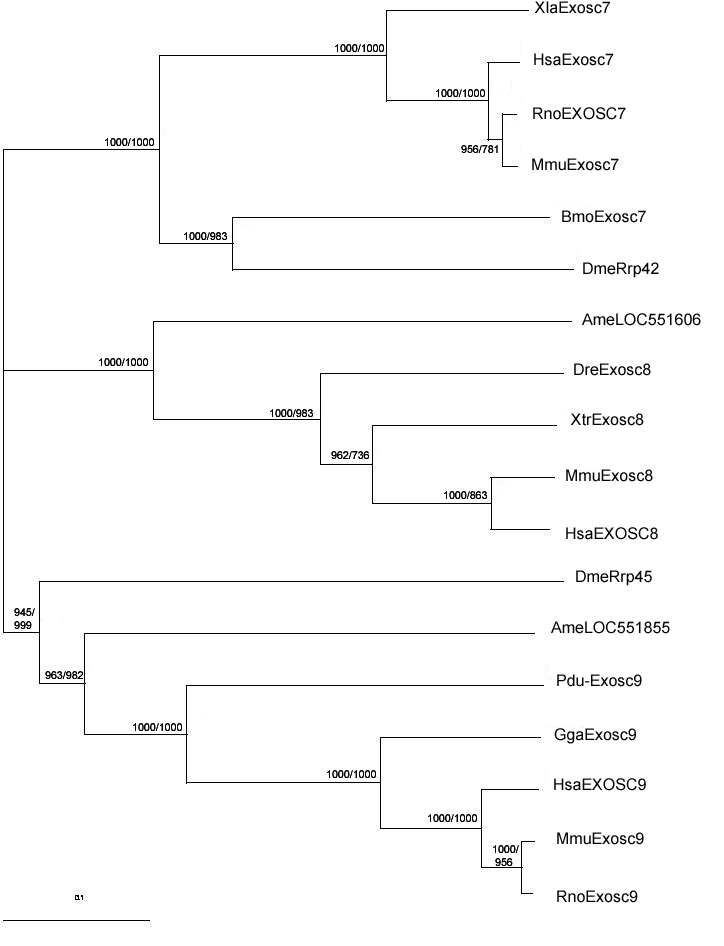


Hui_Fig. S12b
